# A droplet reactor on a super-hydrophobic surface allows control and characterization of amyloid fibril growth

**DOI:** 10.1038/s42003-020-01187-7

**Published:** 2020-08-20

**Authors:** Peng Zhang, Manola Moretti, Marco Allione, Yuansi Tian, Javier Ordonez-Loza, Davide Altamura, Cinzia Giannini, Bruno Torre, Gobind Das, Erqiang Li, Sigurdur T. Thoroddsen, S. Mani Sarathy, Ida Autiero, Andrea Giugni, Francesco Gentile, Natalia Malara, Monica Marini, Enzo Di Fabrizio

**Affiliations:** 1 SMILEs Lab, Physical Science and Engineering (PSE) and Biological and Environmental Science and Engineering (BESE) Divisions, King Abdullah University of Science and Technology, Thuwal, 23955-6900 Saudi Arabia; 2 High-Speed Fluids Imaging Lab, Physical Science and Engineering (PSE) Division, King Abdullah University of Science and Technology, Thuwal, 23955-6900 Saudi Arabia; 3 Clean Combustion Research Center, Physical Science and Engineering (PSE) Division, King Abdullah University of Science and Technology, Thuwal, 23955-6900 Saudi Arabia; 4 Istituto di Cristallografia – Consiglio Nazionale delle Ricerche (IC-CNR), Via Amendola 122/O, 70126 Bari, Italy; 5 Department of Physics, Khalifa University, P.O. Box: 127788, Abu Dhabi, UAE; 6 Department of Modern Mechanics, University of Science and Technology of China, Hefei, Anhui 230026 China; 7 Molecular Horizon, Bettona, Italy; 8 National Research Council, Institute of Biostructures and Bioimaging, Naples, Italy; 9 Department of electrical Engineering and Information Technology, University Federico II, Naples, Italy; 10 BIONEM lab, University Magna Graecia, Campus Salvatore Venuta, Viale Europa, 88100 Catanzaro, Italy; 11 Materials and Microsystems Laboratory, Department of Applied Science and Technology, Politecnico di Torino, 10129 Torino, Italy

**Keywords:** Lab-on-a-chip, Protein folding, Raman spectroscopy, Biopolymers in vivo

## Abstract

Methods to produce protein amyloid fibrils, in vitro, and in situ structure characterization, are of primary importance in biology, medicine, and pharmacology. We first demonstrated the droplet on a super-hydrophobic substrate as the reactor to produce protein amyloid fibrils with real-time monitoring of the growth process by using combined light-sheet microscopy and thermal imaging. The molecular structures were characterized by Raman spectroscopy, X-ray diffraction and X-ray scattering. We demonstrated that the convective flow induced by the temperature gradient of the sample is the main driving force in the growth of well-ordered protein fibrils. Particular attention was devoted to PHF6 peptide and full-length Tau441 protein to form amyloid fibrils. By a combined experimental with the molecular dynamics simulations, the conformational polymorphism of these amyloid fibrils were characterized. The study provided a feasible procedure to optimize the amyloid fibrils formation and characterizations of other types of proteins in future studies.

## Abstract (web summary)

Zhang et al present an integrated real-time imaging and flow field control platform based on water droplet evaporation on super-hydrophobic substrate (SHS) to enable amyloid fibril aggregation. They apply this methodology to observe structural polymorphism in PHF6 peptide and full length Tau441.

## Introduction

Protein misfolding causing assembly and amyloid fibril aggregation is a huge matter in many human neurodegenerative diseases^1,2^: in particular Tau protein which is one of the major players in Alzheimer disease (AD), Pick’s disease, Chronic Traumatic Encephalopathy (CTE), Parkinson’s disease, and Progressive Supranuclear Palsy (PSP)^3^. Molecular structures characterizations of these amyloid fibril aggregations are extremely important to understand the pathogenesis of these diseases for new therapies development^2^. However, these protein amyloid fibrils are difficult to form without using additional chemical cofactors, which hampers further molecular structure characterizations in vitro, especially for the full length (441 amino acids) Tau protein^4,5^. In recent years, increasing evidences indicated that the fluid flow is one of the most important non-chemical factors causing protein aggregation^6–8^. In spite of many efforts have been reported in this field by several teams, the method that could provide real-time direct monitoring of this phenomenon is still undiscovered and its application in the in situ bio-medical molecular structure characterization is minor^8–15^.

Herein, we demonstrated a methodology and an integrated real-time imaging and flow-field control platform based on water droplet evaporation on super-hydrophobic substrate (SHS) to produce in vitro amyloid fibril aggregation for in situ molecular structure characterizations. With this methodology and platform, we were able to produce the aggregation of amyloid fibrils without the aid of any aggregation cofactor for molecular structure characterization, which is not straightforward, especially in the case of full-length Tau protein.

SHS based on micro-pillar arrays (Supplementary Note 1 and Supplementary Fig. 1) had been widely used in single-molecule manipulation and detection of various macromolecules, including DNA and proteins, by taking advantage of the molecules accumulation and assembly under Marangoni convective flow and drop receding mechanism under evaporation^16–22^. Given the inherent convective flow in the liquid droplet, SHS should be an ideal platform to mimic and analyze the protein molecular behavior under continue flow in real time. Although many theoretical and experimental efforts have been made to study the convection phenomenon in liquid droplet, the experimental control and optimization of the convective flow for real-time molecule manipulation and analysis is absent, due to experimental obstacles, in particular, the droplet imaging control during the aggregation process^23–26^.

We built a light-sheet imaging system (Supplementary Note 2 and Supplementary Fig. 2) to study the real-time hydrodynamic behavior of protein molecules under confined convective flow in a droplet on SHS with the task to obtain well-ordered fibrils where information on protein structure can be determined through Raman and X-ray diffraction methods. By taking advantage of real-time droplet imaging, the convective flows in droplet were optimized with temperature control. The very first direct visual evidence was acquired and showed that the convective flow can spatially confine the protein molecules present in the droplet and therefore enhance the assembly into amyloids or fibrils aggregates. In addition, due to the advanced design of the SHS platform, the molecular structures of formed fibrils aggregates were self-suspended aiding the analysis by confocal Raman spectroscopy, two-dimensional X-ray diffraction (2D-XRD), small-angle X-ray scattering (SAXS) and wide-angle X-ray scattering (WAXS).

We applied our methodology and platform in the analysis of Tau protein isoform 2N4R (Tau441) and its peptide fragment PHF6, widely recognized to be involved in amyloid-type aggregation in Alzheimer’s disease^27–29^. This experimental platform allowed the formation of amyloid fibers from PHF6 and Tau441 that presented several degrees of conformational polymorphism upon change of temperature gradient, as will be discussed in the following sections. Finally, we applied molecular dynamics (MD) simulation on PHF6 and Tau441 systems in order to derive detailed insights of the fibril properties^30–33^.

**Fig. 1:**
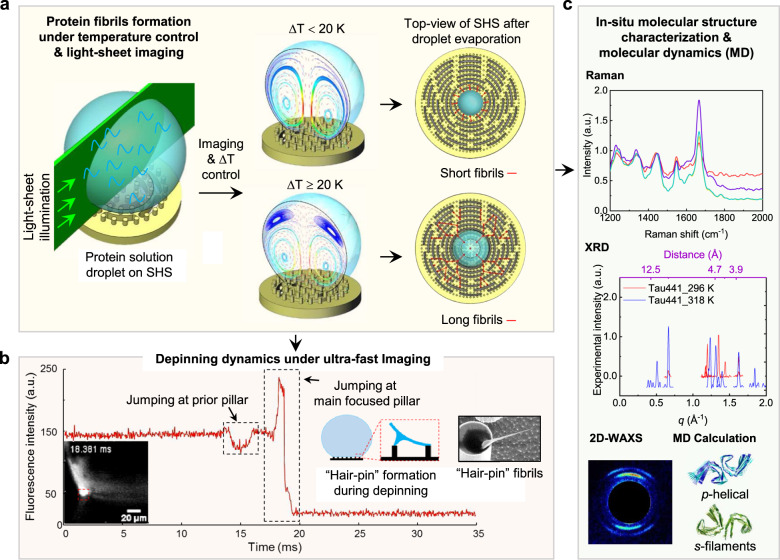
Scheme of the whole process from protein fibrils formation in droplet with confined convective flow field control and study of droplet contact line depinning dynamics to protein fibrils molecular structure characterizations. **a** Protein fibrils formation with temperature gradient (Δ*T*) controlled confined convective flow field in droplet on SHS. **b** Droplet contact line depinning dynamics study with ultra-fast imaging. **c** In situ molecular structure characterizations of protein fibrils with Raman, XRD, 2D-WAXS, and molecular dynamics (MD) calculation.

The schematic of the whole process is briefly shown in Fig. 1.

## Results

### Protein amyloid fibrils assembling and growing driven by confined convective flow

**Fig. 2:**
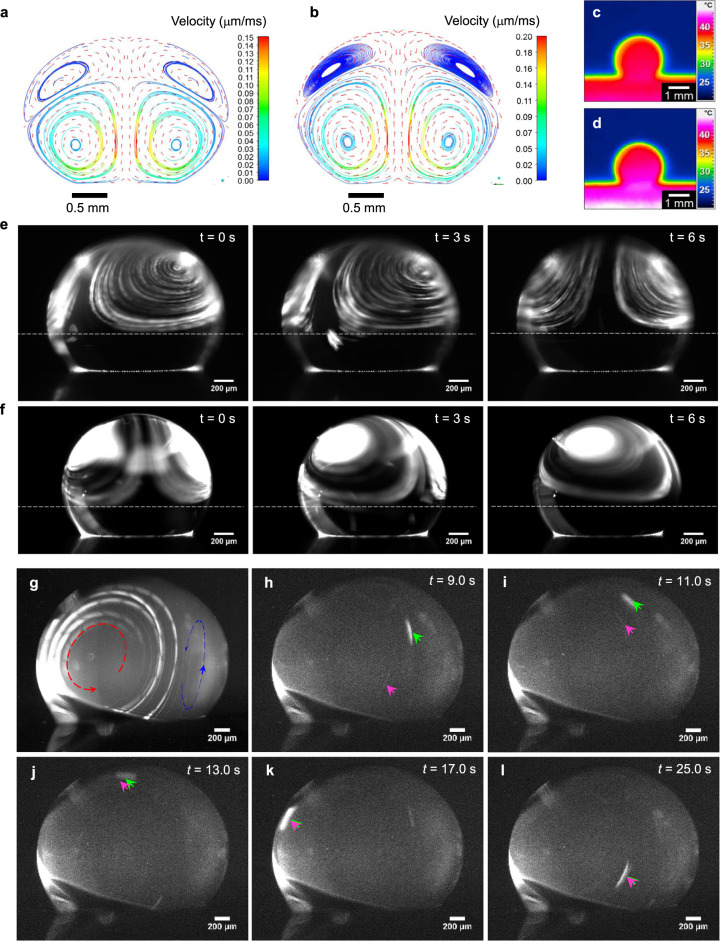
Simulated and experimental convective flow in water droplet on SHS for molecules driven. **a** Simulated convective flow field in droplet driven by buoyancy force at Δ*T* = 20 K and at **b**, Δ*T* = 25 K. **c** thermal imaging of water droplet on hotplate at Δ*T* = 20 K and at **d**, Δ*T* = 25 K. **e** Experimental convective flow at Δ*T* = 20 K and at **f**, Δ*T* = 25 K. **g** Amyloid fibrils driven by confined convection flow in droplet at Δ*T* = 25 K. The closed loops indicate the total circulation of in droplet and the red arrow indicates the circulation direction. In general, the convection loop is pairwise, as we showed previously. Here one of the loop is observed clearly, and the paired one is on the “back-side” of the droplet as shown with blue line. **h**–**l** Real-time tracking of a typical amyloid fibrils assembling and growing driven by confined convective flow. Time stamps indicate the different frames of the imaging flow.

To study the flow field driven protein aggregation in real-time, a confined and steady flow is required. Although several mechanic stirring based systems had been reported, none of them showed the ability to perform real-time investigations^12,34,35^. And according to our previous research about droplet on SHS, the inherent convective flow in evaporating droplet should be a highly potential candidate as fiber aggregation driving force^16,18,36^. Here we produced a confined and steady convective flow (Fig. 2) driven by temperature gradient, Δ*T* (i.e., a water droplet with given size sitting on a heated substrate, Δ*T* = *T*_1_−*T*_0_, *T*_1_ is the temperature of hot substrate, *T*_0_ is ambient temperature, Supplementary Note 3).

Unlike Marangoni convection that drives mass transfer from droplet edge to center, at the interface of air, water and the solid substrate, and impacts the substrate (Supplementary Note 1, Supplementary Fig. 6 and Supplementary Movie 1), the buoyancy force, depending on temperature gradient, drives loop fluxes and objects floating away from the bottom (Fig. 2a, b). This effect, pursued in this work, is central in driving the fibril aggregation, in fact the top loop fluxes (Fig. 2a, b) are effective for generating a steady-state loop and avoid the impact of proteins onto the substrate before the end of the evaporation process. The top loop fluxes generate a continuous convective flow far from the substrate without any deposition of the solute. Indeed, in our experimental conditions, this happened when the temperature gradient, respect to the environment, reached or overcame Δ*T* = 20 K (Fig. 2c–f). The convective flow pattern was changing significantly compared to Δ*T* < 20 K (Supplementary Note 3, Supplementary Fig. 6 and Supplementary Movie 1). Two stable and steady symmetric convective flows, on the top of the drop, were obtained (Fig. 2e, f, and Supplementary Movie 2). These flows are lifted from the substrate. Meanwhile, compared to the conditions at Δ*T* < 20 K (Supplementary Note 3, Supplementary Fig. 6 and Supplementary Movie 1) the confined convective flow driven by the buoyancy force is quite stable and the airflow does not affect the symmetrical vortexes but only drives their rotation about the vertical axis of the droplet (Fig. 2e, f, from *t* = 0 s to *t* = 6 s). The discontinuous convective flow at Δ*T* < 20 K is not effective for fibril aggregation, because it intercepts the substrate and deposits the molecules on it, preventing the fibril aggregation that happens only after longer looping time. In addition, although the simulation suggested the presence of four convective vortexes in the droplet with the two convective vortexes on top of the droplet and two convective vortexes at the bottom, the experimental data showed only two strong vortexes on the top side of the droplet. Since the two strong top vortexes attracting most of the fluorescent beads, the fluorescent in the bottom part of the drop becomes so week that optical image becomes dark, therefore, only the top part of the droplet maintains a steady state molecular concentration and contribute to fiber aggregation can be imaged by the light-sheet microscope.

We applied this confined convective flow to manipulate and force the protein aggregation. We started the experiment by dropping the pre-formed amyloid fibrils solution from hen egg weight lysozyme (HEWL) with fibers of ~5 nm thickness as obtained from AFM measurement (Supplementary Note 4 and Supplementary Fig. 10) on SHS. The real-time images driven by confined convective flow were acquired (Fig. 2g). Clearly, the amyloid fibrils aggregation is driven continuously by the pairwise confined convection loops in the droplet. The molecule trajectories are not disturbed by the substrate, thus forming a persistent convection loop. In addition, by taking advantage of the real-time imaging system, two representative fibril aggregates (Fig. 2h–l, the green and magenta arrowed), were tracked in the convective flow. Initially, these two different sized fibrils migrate independently at large relative distance (Fig. 2h, Supplementary Note 5, Supplementary Movie 3 and Supplementary Fig. 11). Due to the convective flow loops, these two fibrils are driven closer and closer (Fig. 2i, j) until they aggregate together and form one single larger assembly (Fig. 2k, l).

Although several previous researches provided experimental evidence of the amyloid fibrils formation induced by the shear-flow, these results were based on the measurement of fluorescence intensity collected from the amyloid aggregates^10–12^. The size factors of amyloid fibrils aggregates were evaluated by their fluorescence intensities. In addition, since the shear force was produced by a mechanic-based approach, the study of amyloid fibrils stabilities under high shear-rate (300 s^−1^) was possible. Compared to these researches, we produced the shear-force with a confined convective flow in droplet, where its moderate shear-rate (<100 s^−1^)^37^ is still responsible for the amyloid fibrils growth process, as we observed in our experiments. Unlike the indirect measurements based on fluorescence intensities, we provide a direct imaging of the protein aggregation driven by a droplet convective flow.

This is the first direct evidence, to our knowledge, in real-time and at the single fibrils level, of amyloid fibrils aggregation and growing, driven by a confined convective flow.

### Morphology of amyloid fibrils deposition

**Fig. 3:**
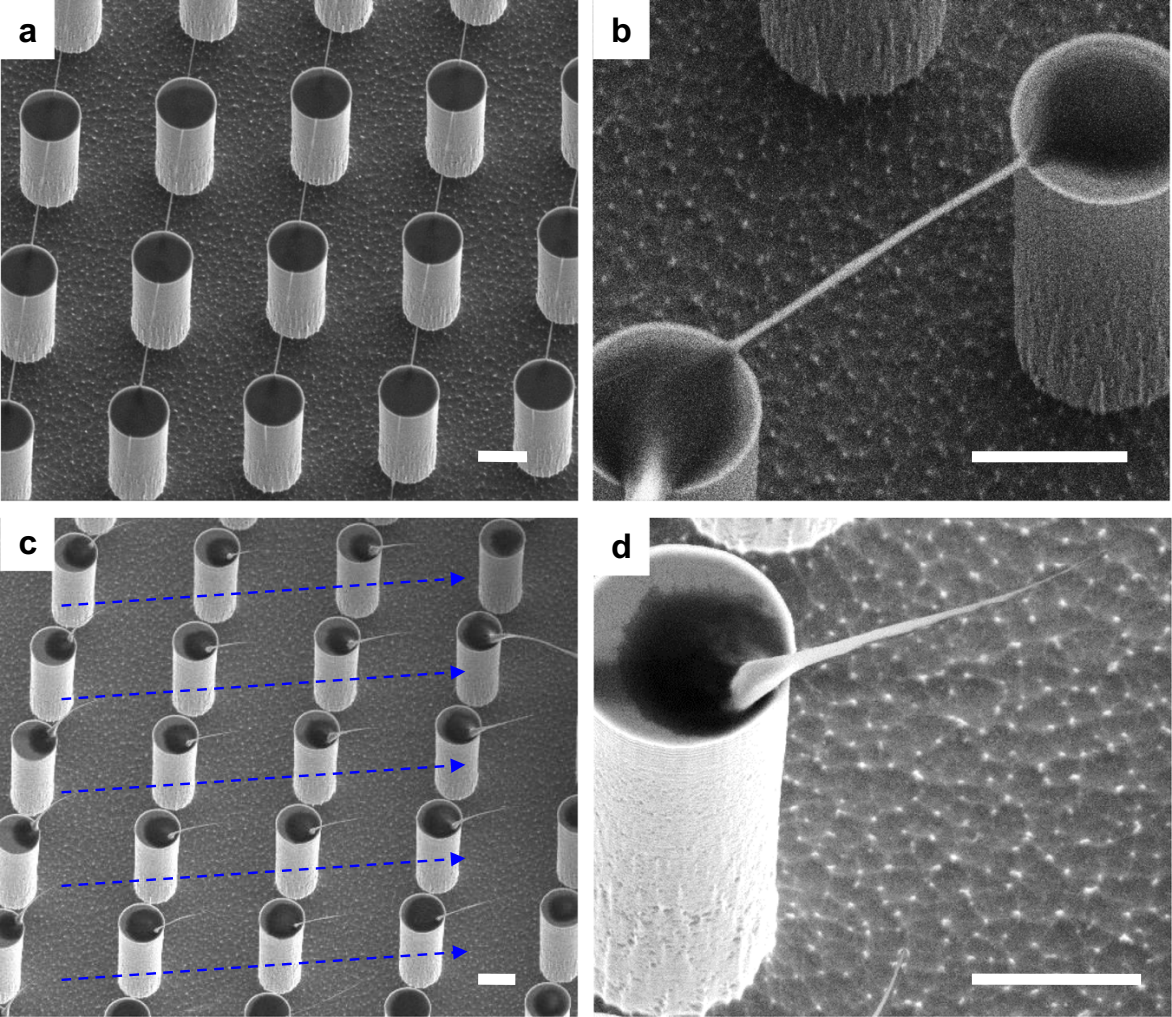
SEM images of final amyloid fibrils depositions on SHS after the drop evaporation. **a**, **b** Suspended and stretched amyloid fibrils with different size. **c**, **d** “Hair-pin” morphology formed on the top of micro-pillars. Scale bars are 5 μm.

Due to the steady motion in the convective flow, the final deposited amyloid fibrils, after drop evaporation and suspension on SHS, are much larger than their initial size (Fig. 3a, b). The SEM images show that these fibrils suspended between micro-pillars are around 100 nm diameter, which is about 20 times larger than their initial size (~5 nm). Moreover, besides the common fibrils suspended between micro-pillars, lots of “hair-pin” structures formed, at first evaporation suspension stage, on the top of micro-pillars and observed (Fig. 3c, d). We analyzed and imaged also these structures because they can be of future interest for structural studies of protein aggregation. Unlike the suspended amyloid fibrils with a uniform thickness along the elongation axis, the “hair-pin” shows a very different diameter size from its tip down to the root of the “hair-pin” (Fig. 3d). The morphology of the final depositions are highly relying on the contact pinning line and on the drop sliding-de-pinning process^16^. The capillary force between the droplet and micro-pillars during the contact line and de-pinning is an important factor to align the amyloid fibrils between pillars to form the highly oriented suspended fibrils^36^. The uniform “hair-pins” orientation indicates the fluidics direction during the contact line de-pinning process (blue arrows in Fig. 3c), and is in agreement with the droplet de-pinning mechanism we could image by fast camera, as shown in the next paragraph.

### Contact line de-pinning dynamics

In general, a drop deposited on a surface evaporates in two distinct evaporation modes: constant contact angle (CCA) mode and constant contact radius (CCR) mode, and the pinning de-pinning mode can be considered as a transition between CCA and CCR^38^. In our case, the contact line defines also the de-pinning contour on the micro-pillars. On the contrary, the pinning contour defines the new contact line after one step of drop sliding caused by the droplet evaporation. The CCA to CCR mode transition was reported in our earlier research^16^. Although several numerical and experimental efforts have been reported to study the pinning and de-pinning process, the real-time dynamic process is difficult to describe analytically^38–42^.

**Fig. 4:**
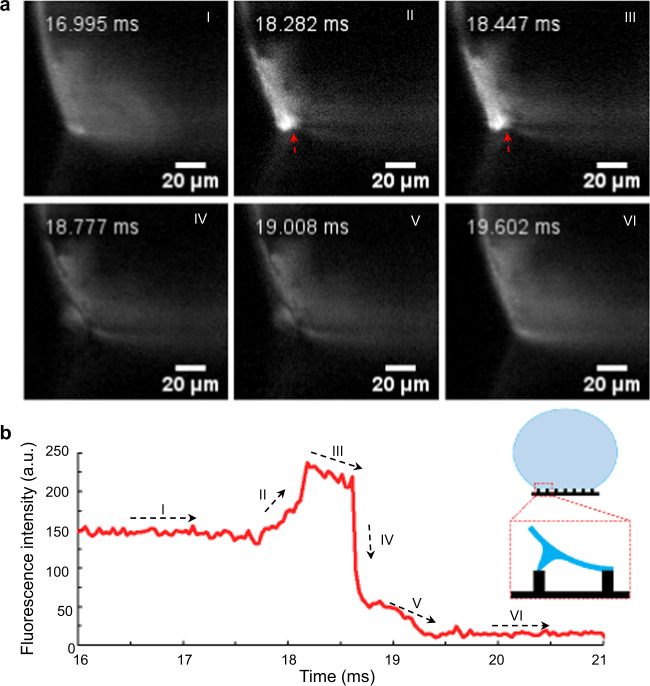
Images of the droplet contact line receding on micro-pillar arrays with ultra-fast imaging technique. **a** Six key steps during the depining process. I, steady-state before depinning; II, transient-state during depinning by forming stretched capillary bridge between outer pillars and droplets; III, capillary bridge stretching and moving; IV, capillary bridge break and disappear, contact line pinning to new area; V, disturbance from posterior pillar; VI, steady-state after depinning. **b** Fluorescence intensity tracking corresponding to the six processes (I–VI) and the inner schematic drawing of the capillary bridge. The fluorescence images are acquired with an ultra-fast camera (Phantom V2511) at 30,000  fps by Leica lens (2.0 × 9.2 × 1.6×).

To overcome this theoretical hurdle, we applied the ultra-fast imaging technique with 30,000 frame-per-second (fps) in order to study the de-pinning process. In particular, we were interested to elucidate the micro-capillary bridge formation between pillars and its dynamics in real-time (Fig. 4a and Supplementary Movie 4). We analyzed the fluorescent signal coming from microbeads dissolved in the droplet exclusively for this task. The detailed analysis of fluorescence intensities at the contact area (Fig. 4b) indicates a six-step evolution of the de-pinning dynamics. Step I: steady-state before de-pinning. Step II: transient-state during de-pinning by consequent formation of stretched micro-capillary bridge between outer pillars and droplets (red arrow in frame II of Fig. 4a). Due to the stretching, a local dehydration caused an increase of the solute concentration that gives a significant increase of fluorescence signal. Step III: micro-capillary bridge stretching and drop sliding (red arrow in frame III of Fig. 4a). In this time frame, a neck is forming, due to Plateau-Rayleigh instability of the liquid, which is responsible for the “hair-pin” shaped morphology reported in Fig. 3c, d^43,44^. As a consequence, the fluorescence intensity decreases slightly. Step IV: micro-capillary bridge breaks and disappears, and a new contact line pinning is formed on the proximal inner pillars. Therefore, the fluorescence intensity decreases dramatically. Step V: the de-pinning induces oscillation and disturbance from back drop boundary, by producing a moderate variation of fluorescence intensity. Step VI: the system reaches a new pinning steady state.

The dynamics of the amyloid fibrils’ depositions on SHS clarified the fibrils growth process. Besides the temperature, the de-pinning process induces the various fibrils morphologies and it is worth to ask: is the depinning responsible for one of the mechanism that generates the polymorphism^45,46^? To answer this question, we investigated the molecular structures of hair-pin and suspended fibrils with XRD and Raman spectroscopy.

### Molecular structure characterization of amyloid fibrils on SHS

2D-XRD and confocal Raman spectroscopy were used to characterize the molecular structures of Lysozyme amyloid fibrils on SHS (Supplementary Notes 6 and 7).

**Fig. 5:**
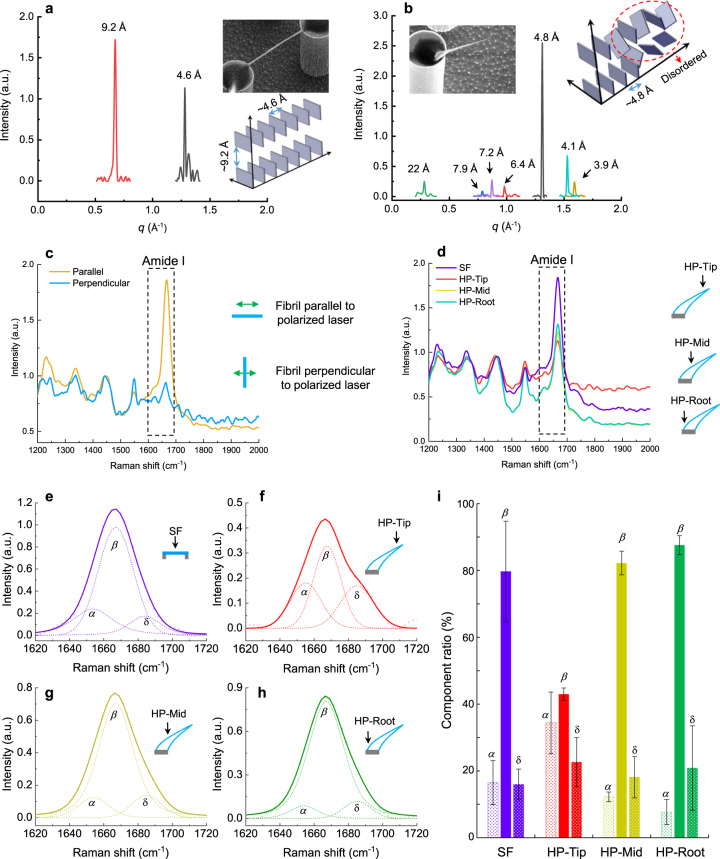
Molecular structure characterizations of lysozyme amyloid fibrils. **a** XRD characterization of lysozyme amyloid fibrils suspended between two pillars. **b** XRD characterization of hair-pin (HP) type lysozyme amyloid fibrils. **c** Polarized Raman spectroscopy of SF. Parallel and perpendicular indicate the fibril is parallel and perpendicular (right panel diagrams) to the incident laser’s polarization respectively. **d** Polarized Raman spectroscopy of SF and HP type lysozyme amyloid fibrils at different positions (tip position, HP-Tip; middle position, HP-Mid; root position, HP-Root). **e**–**h** peak fitting of the measured Raman spectroscopy corresponding to (**d**). *α*, *α*-helix structure; *β*, *β*-sheet structure, *δ*, disordered structure. **i** Calculated component ratio of the three main structures (*α*, *β*, and *δ*) in SF and different positions of HP based on the Raman peaks fitting in (**e**–**h**). Error bars indicated the mean ± standard deviation from three different measurements.

As in previous reports by many groups, the 2D-XRD of amyloid fibrils show two distinctive patterns at ~4.7 Å and 6–11 Å, which correspond to the hydrogen bond spacing between inner- and inter-strands in *β*-sheets, respectively^47^. Here, in our experiments, our XRD pattern contains spots which corresponds to 4.6 Å and 9.2 Å (Fig. 5a), which agree with what expected for amyloid fibrils (Supplementary Note 6). In addition, unlike the general 2D-XRD fiber patterns, which are arched and diffused at around 4.7 Å and 6–11 Å, a diffraction pattern with spots was acquired from our sample (Supplementary Note 6 and Supplementary Fig. 12). In the sample there are single crystals with these lattice spacings. On the other hand, the 2D-XRD patterns from the depositions with “hair-pin” morphology is quite different from the suspended *β*-sheet amyloid fibrils (Fig. 5b, Supplementary Note 6 and Supplementary Fig. 12d). Besides the typical *β*-sheet patterns at 4.8 Å, we find several peaks arising around 4, 7, and 22 Å. We think that this might be caused by two possible reasons. First, it may indicate that there are some different secondary structures involved in the “hair-pin” morphology. Second, since the “hair-pins” orientations are different from general suspended fibrils (Fig. 3d), the additional peaks might be induced by modifications during the measurements. Therefore, additional structure analysis on a single suspended amyloid fibrils and single “hair-pin” is necessary. However, due to the instrumental limitations of the 2D-XRD, it is impossible to resolve the structure differences from single fiber and single “hair-pin”.

Hence, additional analysis on a single suspended amyloid fibrils and single “hair-pin” were performed by confocal Raman spectroscopy (Supplementary Note 7 and Fig. 5c–i).

The suspended amyloid fibrils between micro-pillars on SHS show a significant anisotropic Raman scattering at the Amide I region (1600–1690 cm^−1^) under incident linear polarized laser with two orthogonal laser polarization measurements (parallel and orthogonal respect to hair pin/fiber axes) (532 nm) due to the alignment of hydrogen bonds along fibrils axis (Fig. 5c), in agreement with our previous reports^48,49^.

As we mentioned before, the “hair-pin” morphology is different from its tip down to the root, therefore, the Raman signal of “hair-pin” at different positions (i.e., at tip, middle and root) was measured (Fig. 5d). Since the Raman intensities are proportional to the number of molecules, in comparison with the suspended amyloid fibrils, the Raman signals of hair-pins at 1667 cm^−1^, which is corresponding to the *β*-sheet structure, are much lower, especially at their tip (HP-Tip) position. Since the peak at Amide I region is a convolutional signal from three components, *α*-helix (~1650 cm^−1^) structure, *β*-sheet (~1670 cm^−1^) structure and disordered (*δ*, ~1690 cm^−1^) structure^50^, to understand these components in detail, quantitative peak fitting analysis of the Amide I region was performed (Fig. 5e–h). Three peaks corresponding to *α*-helix structure, *β*-sheet structure and disordered structure were fitted at 1654, 1667 and 1684 cm^−1^, respectively (Fig. 5e–h). The percentage ratio of these three components were calculated from their fitted peaks areas (Fig. 5i). In suspended amyloid fibrils structures, the percentage ratio of these three components are signed as 16.5 ± 6.5, 79.7 ± 15, and 15.0 ± 4.6 respectively, *β*-sheet structure is the major component, in agreement with the XRD pattern. On the other hand, for the “Hair-pin” structures, the percentage ratio of *β*-sheet at middle and root position are 82.2 ± 3.5 and 87.6 ± 2.8, which are higher than the ratio in suspended fibrils. We believe this is the consequence of stretching directional effect from capillary force during contact line de-pinning, as we discussed in last paragraph. Hence, we believe that the anisotropic capillary force in the micro-bridge not only arranges the molecules to assembling along the bridge which induced a highly oriented *β*-sheet, but also induces a reorganization of part of molecules in *α*-helix and disordered structures into *β*-sheet structure. In addition, at the tip position of “hair-pin”, although the *β*-sheet structure still is the major component, its ratio is decreased to 43.0 ± 1.9%, meanwhile the ratios of *α*-helix and disordered structures are increased to 34.4 ± 9.2% and 22.6 ± 7.4%, respectively. Apparently, this is the consequence of the capillary bridge breaking. Therefore, considering the whole picture, the secondary structures of the “hair-pin” morphology is more complex than suspended fibrils, probably due to its transient formation regime (highly out of equilibrium), as confirmed by the XRD measurements.

Hence, we confirmed that the de-pinning process modeled the “hair-pin” morphology outwardly, but more importantly, caused ununiform and more complex secondary structures at molecular level, i.e., induced the polymorphism in the lysozyme amyloid fibrils. Since these polymorphisms were reported in many in vivo conditions (in general, in vivo have much more complex flow field conditions)^51^, we believe the extra flow field is an important factor that induces the polymorphisms during the amyloid fibrils formation. Indeed, as specific molecular conformations are preferred in crystallization processes because of the forces present in the crystals^46^, here forces due to the extra flow field cause the presence of local polymorphisms.

### Amyloid fibrils formed from PHF6 on SHS and molecular structures characterizations

**Fig. 6:**
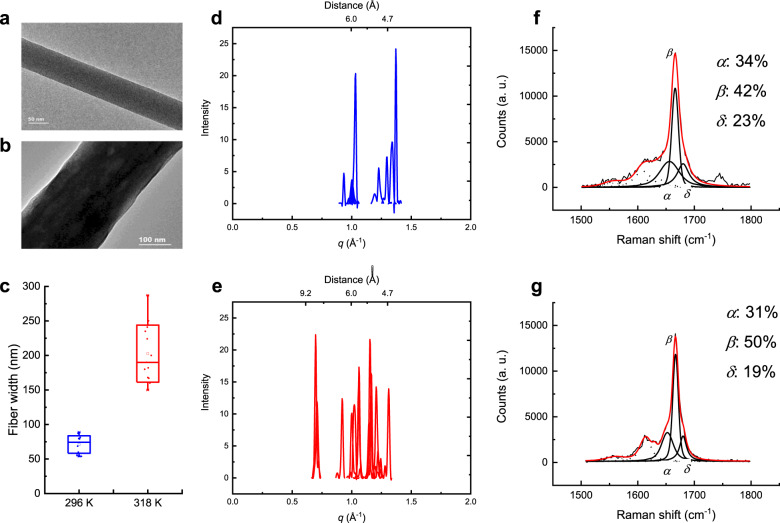
Prepared PHF6 fibrils on SHS and characterized by TEM, XRD, and Raman spectroscopy. **a**, **b** TEM image of PHF6 fibrils formed at 296 K (Δ*T* = 0 K) and 318 K (Δ*T* = 22 K). **c** Width measurements of fibrils formed at 296 K and 318 K. **d**, **e** 2D XRD of PHF6 fibrils formed at 296 and 318 K. **f**, **g** Polarized Raman spectrum (parallel measurements) with cure fitting of PHF6 fibrils formed at 296 and 318 K.

We applied our sample preparations to PHF6^52^ and followed drop solution-evaporation on SHS (Fig. 6). Due to the convective induced molecular growth and aggregation in the droplet, the fibrils formed at 318 K have much larger size compare with the fibrils at 296 K (Fig. 6a, b). With the measurements of various fibrils, the quantified data show that the width of the fibrils formed at 296 K is 71 nm in average, while the width of fibrils formed at 318 K is 202 nm in average (Fig. 6c). Although the width distribution at 318 K is wider than 296 K, these fibrils have much larger average size. Then we measured the 2D-XRD and Raman spectrum of these fibrils. The XRD measurements of fibrils formed at 296 K is shown in Fig. 6d. The peaks centered at 4.7 Å indicate the typical *β*-sheet structures. And interestingly, a more detailed analysis shows that there are three peaks at 4.6, 4.7, and 4.8 Å (Table 1). Since the XRD measurement area covered many fibrils, we think that these peaks indicate the slight differences between various fibrils. In contrast, except for the peaks at about 4.7 Å, the XRD measurements of the fibrils formed at 318 K (Fig. 6e) showed additional peaks at 5.4 and 9.0 Å. The 5.4 Å spacing should correspond to the *α*-helix structure, while, the 9.0 Å may indicate the inter-layer spacing of *β*-sheet^53^. Nevertheless, several peaks around 6.0 Å are unknown, therefore, more analysis with Raman spectroscopy was performed. These fibrils have sensitive response to laser polarization (Supplementary Note 8 and Supplementary Fig. 13) indicating their well-organized *β*-sheet, as we discussed previously. More detailed analysis was performed with peaks fitting (Fig. 6f, g). The fibrils show increasing of *β*-sheet secondary structure (at about 1667 cm^−1^) at 318 K (Fig. 6). Moreover, the FWHM of this peak is slightly lower at 318 K (12 cm^−1^) than at 296 K (14 cm^−1^), indicating a higher fiber order. Notably, by comparing the ratio of the intensity values at 1667 cm^−1^ peak (polarization along the longitudinal axis), with the intensity with orthogonal polarization, we found that at high temperature, 318 K, this ratio is 17% lower. This could indicate a general higher order of the fibrils structure in the high temperature case, especially in the orthogonal direction of the laser polarization. And the results agree with MD calculations (Supplementary Note 11).

**Table 1:** XRD spacing of amyloid fibrils formed from PHF6 peptides and full-length Tau protein (Tau441) at 296 K and 318 K.

Structures	Spacing from different samples (Å)			
	PHF6		Tau441	
	296 K	318 K	296 K	318 K
*β*-sheet	4.6 4.7 4.8	4.8 8.9 9.0	4.4 4.6 9.5	4.5 4.7 4.8 9.4 10.2 12.3
*α*-helix	–	5.4 5.4 5.5	5.4	5.5
Un-known/disordered	5.1 6.1 6.2 6.3 6.7	5.1 5.2 5.9 6.1 6.3 6.8	3.9 5.2 5.3	3.9 5.1

### Tau441 amyloid fibrils on SHS and molecular structures characterizations

**Fig. 7:**
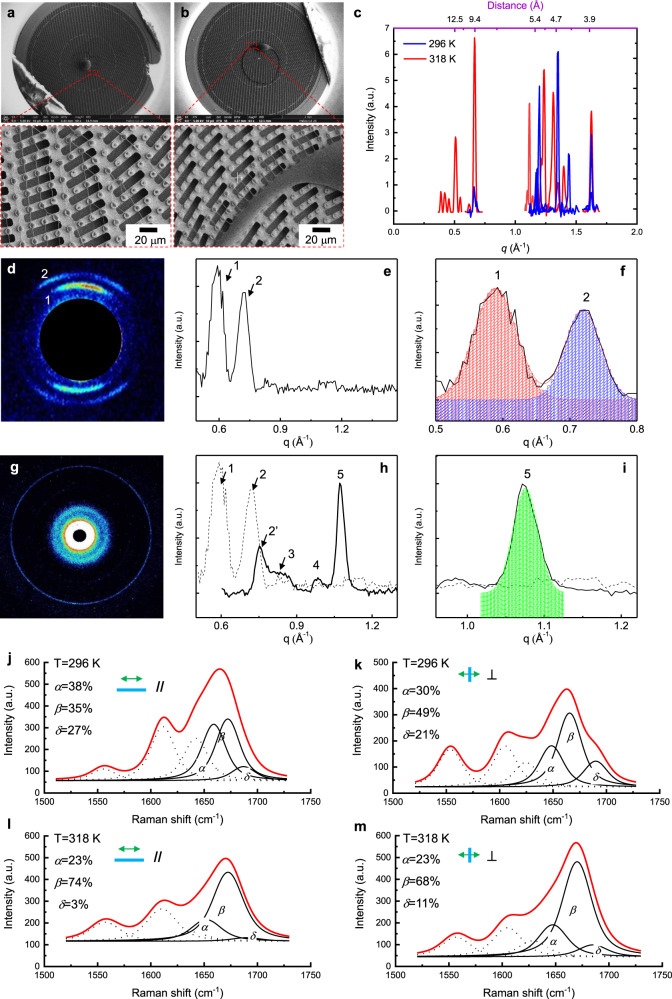
Prepared Tau441 fibrils on SHS and characterized with XRD, WAXS, and polarized Raman spectra. **a** Tau441 fibrils formed at 318 K (Δ*T* = 22 K) and **b**, Tau441 fibrils formed at 296 K (Δ*T* = 0 K). **c** Experimental (red and blue peaks) and calculated amyloid fibrils (green peaks) XRD of suspended fibrils formed form Tau441. Tau441_296 K and Tau441_318 K indicate the fibrils formed from Tau441 at room temperature (296 K) and high temperature (318 K), respectively. **d** 2D WAXS of Tau441, collected at 3 cm in the point marked as (1) in Supplementary Fig. 14, shows a fiber pattern (anisotropic distribution of intensity with lobes). **e** 1D WAXS, after calibration and folding of the corresponding 2D WAXS pattern in (**d**), shows two main peaks, marked as 1 and 2, plus additional faint peaks. **f** Gaussian fitting of the peaks 1 and 2. **g** 2D WAXS of Tau441, collected at 10 cm, across the area marked as 3 in Supplymentary Fig. 14. **h** 1D WAXS, after calibration and folding of the corresponding 2D WAXS pattern in (**g**), shows the peaks marked as 2’, 3, 4 and 5. **i** Gaussian fitting of the peak 5. Results of the fitting in Supplementary Notes 10. **j**–**m** Raman spectra of Tau full length both at 296 K and 318 K. Deconvolution of Amide I indicate the components *α*-helix (*α*), *β*-sheet (*β*) and disordered structure (*δ*). **j**–**k** Deconvolution of Amide I of Tau441 Raman spectrum at 296 K and laser polarization parallel to fibrils (//) and laser polarization perpendicular to fibrils (⊥). **l**–**m** Deconvolution of Amide I of Tau441 Raman spectrum at 318 K and laser polarization parallel to fibrils (//) and laser polarization perpendicular to fibrils (⊥).

Tau441 full-length protein is an intrinsically disordered protein and has never been studied in vitro in amyloid fibrils form because of the difficulty in the experimental formation of its amyloid fibrils form^54^ (Supplementary Note 4 and Supplementary Fig. 10). As we expected, the number of suspended Tau441 fibrils is higher at 318 K (Δ*T* = 22 K) and their length is longer than those at 296 K (room temperature, Δ*T* = 0 K) conditions (Fig. 7a, b). They were characterized with XRD and WAXS (Fig. 7). In the case of Tau fibrils at room temperature (296 K), six relevant diffraction peaks (Table 1) are recognized at 4.4, 4.6, 5.2, 5.3, 5.4, and 9.5 Å from XRD pattern (Fig. 7c). These peaks indicated *β*-sheet (4.4, 4.6, and 9.5 Å) and *α*-helix (5.4 Å) structures which are the main secondary structures in the assemblies. On the other hand, in the case of fibrils formed at high temperature (318 K), except for the typical inner *β*-sheet spacing (4.5, 4.7, 4.8 Å) and a slight shift of *α*-helix (5.5 Å), two important peaks at 10.2 and 12.3 Å are measured (Table 1). According to previous report from a recombinant Tau protein, the peak at 10.2 Å is a typical inter-layer spacing of *β*-sheet, however the peak at 12.3 Å is unclear^53^. To understand the peak at 12.3 Å, we performed MD calculations (Supplementary Note 11) which suggested that the inter-molecular distances between facing subunits, at the interfaces between two lateral Tau441 chains, result approximately 12 Å along both the trajectories (Supplementary Table 10). We attribute these results, even if they are rather fluctuating along the simulations events, to the 12.3 Å XRD peak registered at 318 K. Also, the MD simulations suggest a higher variability of inter-molecular distances respect to the intra-molecular distances (standard deviations in Supplementary Tables 10 and 11, respectively), likely explaining the smaller peak intensity at 12.5 Å of XRD measurement. Indeed, these connections are responsible for the super-molecular architecture which represents a second step of aggregation, weaker than the intra-molecular interactions. MD trajectories suggest that the *s*-filaments (straight filament) proto-filament subunit interface confers greater global stability than that in *p*-helical (paired helical filament) arrangement (Supplementary Note 11 and Supplementary Fig. 19). The simulations could clarify the peak at 12.3 Å of XRD measurements registered only at high temperature, suggesting that the laminar convective flow induces the ordered molecular assembling, and that the fiber, formed at high temperature, preferentially arranges in the more stable *s*-filament morphology. These data confirm again that the amyloid fibrils formed at 318 K have more intact molecular structures.

The 2D-WAXS measurements of the structures formed at 318 K (Fig. 7d–i) showed a typical fiber diffraction pattern (which was never revealed from the XRD measurement, probably because of the limited beam quality of the XRD instrument) with specific peaks (Supplementary Note 10 and Supplementary Fig. 16). These structures are highly ordered *β*-sheet amyloid fibrils^55,56^.  Fig. 7e shows two major peaks, marked as “1” and “2”, plus other faint additional peaks. These two major peaks were fitted by Gaussian function (Fig. 7f) to determine the peak position of 0.51 and 0.64 Å^−1^, which means that the lattice planes distances are of 12.3 ± 0.5 (“1”) and 9.8 ± 0.5 Å (“2”), respectively, in agreement with XRD measurements. The same procedure of data reduction (centering, calibration, 2D-1D folding, and background subtraction) was repeated also for Fig. 7g, resulting in the profile in Fig. 7h. Here, apart for peak “2”, which is slightly moved with respect to the same peak, and it is marked as “2’” in Fig. 7h, additional peaks were measured. The most intense peak, marked as “5”, representing the signature of *β*-amyloid character, is reported in Fig. 7i. Supplementary Fig. 16i summarizes the results of all the diffraction peaks and corresponding distances, measured for Tau441.

Moreover, as we anticipated, the convective flow force field in the droplet could induce a polymorphism of the fibril molecular assembly and a limited variable disorder degree. In order to clarify the degree of disorder of the formed structures, we performed polarized Raman spectroscopy.

The polarized Raman spectroscopy and the relation with the molecular structures of Tau441 fibrils is reported in Fig. 7j–m. In particular, the amount of *β*-sheet (peak at about 1667 cm^−1^) is consistently increased at high temperature at both investigated polarization. At high temperature there is also a decrease of unordered structures (peak at about 1685 cm^−1^) and an increase of *α*-helix secondary structures (peak at about 1655 cm^−1^). Of interest is also the change in the width of the fitted peak for the *β*-sheet component, which is consistently decreasing at 318 K and with longitudinal polarization, again indicating the effect of the laminar flow at higher temperature on the ordered assembly of the Tau441 fibers in the orthogonal direction. This change of behavior is more marked in Tau441 than in PHF6, in agreement with the XRD data (Fig. 6) where the highest diffraction reflection for PHF6 is found at about 9 Å while for Tau441 is at about 12 Å. We found that the ratio of the longitudinal versus the orthogonal polarization intensity area values of the 1667 cm^−1^ is almost half at 318 K than at 296 K. This is an indication of a higher order aggregation in the orthogonal axis achieved only in flow field at 318 K. In addition, the* β* sheet ratio in the fibrils formed at 318 K (74%, Fig. 7l) is two times higher than the fibrils at 296 K (35%, Fig. 7j). The increasing of the *β*-sheet ratio should be induced by the confinement of the disordered component, which percentage decreased from 27% in 296 K condition to 3% in 318 K condition. We can call this confinement phenomenon as *δ*-to-*β* transformation. Again, it indicates that the flow field is playing an important role to produce well-confined amyloid fibrils.

Therefore, for the first time, to our knowledge, we showed experimentally that amyloid fibrils can be produced with full-length Tau441 protein in a droplet reactor without cofactors. With the conjoint Raman, XRD and WAXS analysis, the molecular structures of these fibrils were clarified, including the relative polymorphism.

## Discussion

We developed a methodology and platform to observe, optimize and experimentally control the growth of ordered fibrils of proteins of medical interest by a confined convective flow in droplet on a SHS by real-time study of the dynamics of protein fiber growth. We showed that the ordered fiber aggregation phenomenon is driven by the optimized confined convective flow. The complex growing and deposition mechanisms were recorded both in stationary and fast dynamic conditions showing the very first evidence of protein assembling and growing optimal conditions. The optical imaging based on light-sheet microscopy allowed the understanding of optimal stationary thermo-fluidic growth conditions, the ultra-high-speed imaging technique revealed the fast dynamics of contact line de-pinning during the droplet evaporation and provided an explanation of the morphological diversities of the final deposition construct. The molecular structures of the protein assemblies were analyzed and resolved with XRD, SAXS/WAXS and confocal Raman spectroscopy giving a confirmation of the ordered character of the deposited protein fibrils. The MD study confirmed the structural results deduced by the experiments. It is worth notice that for the first time, we were able to obtain amyloid like aggregation of full-length Tau protein without the aid of any aggregation cofactor, therefore providing a possible protocol for future experimental work for protein structure characterization of Tau proteins and other type of proteins difficult to characterize. With complementary Raman, XRD and WAXS characterization, the molecular structures of these fibrils were inspected, including some polymorphism aspects studied as both a function of non-equilibrium growth conditions (depinning) and a function of temperature gradient (Table 1). From the above analysis, for PHF6 and Tau441 at 296 K and 318 K, and from diffraction data reported in Table 1, it emerges that the additional peaks measured at higher temperature are a manifestation of structural polymorphism. In fact, at higher temperature, we obtained, both for PHF6 and Tau441, additional peaks related to intermolecular order, as explained above, showing that the temperature and flux conditions are responsible for polymorphism. This is an important finding of this work, because it clarifies the wide structural variability found in vitro conditions. These results clearly show the importance of laminar flow in protein aggregation. From a bio-medical point of view this method can therefore, on one side, help in the growth of protein fibrils to be used for further characterization of their molecular structure with classical crystallographic and spectroscopic methods. Besides, it can help to elucidate other processes of aggregation that can be difficult to prove experimentally, such as for example the importance of laminar flow and molecular crowding in the interstitial fluid of the brain, where these amyloid aggregates have their most fatal consequences. With these findings we plan to extend the present experimental platform to a wider category of proteins in order to help in elucidating their unknown structures. These proteins will certainly include all categories of intrinsically disordered proteins given the difficulty in characterizing their full-length structure, either with X-ray crystallography or NMR. Moreover, we could envision that this device will have an application even in the growth of fibrils of membrane proteins (as from our preliminary experimental data for MHC-I protein) embedded in an amphiphilic environment, which is again a difficult task to perform with classical preparation for crystallography methods. In pharmacology, this method could be applied to form ordered fibrils of molecules interacting with their test drug.

## Methods

### SHS design and fabrication

The SHS with micro-pillars array was fabricated based on our previous reports^16^. In brief, the pattern was designed and fabricated as a radial array of pillars with 18 μm radial pitch and each of the pillar having a diameter of 6 μm and height of 10–20 μm on a 4-inch standard Si <100> wafer with 500 μm thickness. In addition, an alternative SHS with micro-pillars and micro-holes array was made for XRD and WAXS measurements. The fabrication combines optical lithography and deep reactive ion etching (DRIE) techniques. We employed 4-inch Si <100> wafer with 50 μm thickness. In the first step, the pillar geometry was obtained by optical lithography with negative resist. A DRIE process (Plasma Lab System 100, Oxford Instr.), was used for the etching of Si substrate obtaining a final pillars height of about 20 μm. After that, the substrate underwent to a second lithography and DRIE to finally create substrate inter-pillar micro-holes. Finally, per-fluor-decyl-trichloro-silane (FDTS) was deposited on the surface by a Molecular Vapor Deposition System (MVD100E, Applied MST). The morphology and the super-hydrophobicity of the substrates were characterized with scanning electron microscopy (SEM, FEI Quanta 600) and contact angle measurements (Supplementary Fig. 1).

Deposited sample on SHS are characterized by SEM or transmission electron microscopy (TEM, FEI Tecnai G2).

### Lab-built LSM for droplet imaging

The customized LSM droplet imaging system (Supplementary Fig. 2) was designed and built based on the OpenSPIM^57^. In brief, a 200-mW 532-nm laser (OXXIUS, France) was aligned to pass-through a beam-expander (two convex lens) and a cylindrical lens (*f* = 50 mm, Thorlabs, USA) to form a laser sheet. The laser sheet width was modulated by a mechanical slit then reflected and focused to a droplet on SHS (Supplementary Fig. 2a). Band-pass filter (610 ± 20 nm, Thorlabs, USA) purified the emitted fluorescence and the images were acquired by a high-speed CMOS camera (ORCA-Flash, HAMAMATSU, Japan) set behind a ×4 microscope objective lens (Plan Fluor, Nikon, Japan) and imaging tube lens (TTL200, Thorlabs, USA) (Supplementary Fig. 2b). In addition, a thermal camera (Vario CAM head Hires 640 G sl, InfraTec GmbH, Germany) was set to map the temperature in droplet with 10 fps and a data correction process (Supplementary Fig. 2a).

In a second set-up, the ultra-fast camera (Phantom v2511, Vision Research, USA) and Leica lens-set (2.0 × 9.2 × 1.6×) were used to investigate the droplet sliding dynamics during the evaporation (Supplementary Fig. 3).

A SHS was placed on the specimen stage, and its temperature was controlled by a thermoelectric effect hot-plate (THE) and lab-written LabVIEW program (Supplementary Fig. 2c).

In order to study the fast sliding transition from proximal pillars, a Melamine resin based micro-bead labeled with Rhodamine (5.0-μm in diameter, Sigma, Switzerland) was dispersed in water and diluted to 5.0 × 10^5^ particles/ml. Then, 6.0 μL of this solution was dropped on the SHS for imaging. The images acquired from CMOS camera were analyzed with single-particle tracking algorithm based on ImageJ^58^.

### Numerical model of convection in droplet and simulation

ANSYS®, Release 19.1 (Fluent solver) is used to solve the governing equations, using the pressure-based finite volume scheme, the pressure-velocity coupling is solved using the PISO algorithm with PRESTO spatial discretization for pressure and Second Order Upwind for Momentum, Density and Energy equations. More details were shown in Supplementary Note 3.

### Protein samples preparation

Pre-formed lysozyme amyloid fibrils were produced starting from a solution of HEWL powder (Sigma) (10 mg/ml) in MilliQ water according to previous reports^49,59^. Full-length Tau (Tau441, 441 amino acids in length) was purchased in lyophilized form (buffer prior lyophilization: 50 mM Tris-HCl pH 7.5, 150 mM NaCl, 0.25 mM DTT, 0.1 mM PMSF) from SignalChem (Richmond, BC, Canada). And PHF6 peptide was purchased as acetyl-PHF6-amide-trifluoroacetate salt (sequence: VQIVYK, PDB file: 2ON9) from Bachem (Bachem AG, Switzerland). The pre-formed HEWL lysozyme amyloid fibrils, PHF6 peptide solution and Tau441 protein solution were characterized with AFM (JPK Nanowizard III and Fig. 10) to confirm their initial state. More details are shown in Supplementary Note 4.

These protein solutions were dropped on the SHS for evaporation with temperature control which was mentioned previous. The residues after droplet evaporation were characterized with SEM/TEM, XRD, Raman spectroscopy, and SAXS/WAXS for morphology and molecular structure analysis.

### XRD measurements

The SHS with micro-holes were used for protein self-aggregation and suspension from a sessile droplet. After drying at same condition, even the substrates alone (with no deposited proteins) were characterized by X-ray diffractometer with 2D detector (D8 Venture, Bruker, USA).To minimize the diffraction signal from substrate, the SHS was glued to stand on a tip vertically and orthogonal to X-ray source (Cu target, 50 kV) perpendicularly (Supplementary Note 6 and Supplementary Fig. 12). The diffraction patterns were acquired by a detector at 40 mm distance with 600 s exposure time.

### Laser confocal Raman measurements

Raman spectrum of the deposited protein fibrils were measured with a laser confocal Raman microscopy (Alpha300 RA, WITec, Germany). The back Raman scattering signal under 4-mW 532 nm laser (Coherent Compass Sapphire Laser) was collected by a ×100 objective lens (Zeiss, EC EPIPLAN NEOFLUAR, 0.9 NA) and then detected by a CCD detector (DU970) at −65 °C. For each measurement at least three spectra were collected, baseline subtracted with a polynomial curve of grade 5, normalized at the peak 1450 cm^−1^, and averaged. Fitting of Amide I peak was performed by using three Voigt functions each centered at 1655, 1668, and 1685 cm^−1^, related to *α*-helix, *β*-sheet and disordered secondary structure of the protein, respectively. More details shown in Supplementary Note 7.

### SAXS and WAXS measurements

SAXS and WAXS data were collected at the X-ray MicroImaging Laboratory (XMI-Lab) which is equipped with a super bright synchrotron class table top X-ray micro-source (Cu Ka, *λ* = 0.15405 nm, 2475 W, rotating anode) coupled, by a multilayer focusing optics (Confocal Max-Flux; CMF 15-105), to a SAXS/WAXS three pinhole Rigaku SMAX-3000 camera (Supplementary Note 10 and Supplementary Fig. 14a).

### MD calculation

MD simulations of PHF6 (50 ns) and Tau441 (two morphologies for 30 ns each) were performed applying the same protocol as shown in Supplementary Note 11.

### Statistics and reproducibility

Our measurements on prepared samples are based on basic data statistics. The multiple (>10) samples with prepared protein fibers are measured by Raman, XRD and WAXS/SAXS for data statistics.

### Reporting summary

Further information on research design is available in the Nature Research Reporting Summary linked to this article.

## Supplementary information

Supplementary Information

Supplementary Movie 1

Supplementary Movie 2

Supplementary Movie 3

Supplementary Movie 4

Description of Additional Supplementary Files

Reporting Summary

## Data Availability

All source data underlying the graphs and charts presented in the main figures are available through Dryad https://datadryad.org/stash/share/UpN68kYKnBxs4z5iPvGo9-HbybXiXtfTD0Kc7KKfwo8.
