# Supplementary material for: A droplet reactor on a super-hydrophobic surface allows control and characterization of amyloid fibril growth
Source: Commun Biol. 2020 Aug 20;3:457. doi: 10.1038/s42003-020-01187-7 (PMC7441408; doi:10.1038/s42003-020-01187-7)
Supplement: Supplementary file 1 — Supplementary Information [file 42003_2020_1187_MOESM1_ESM.pdf]

Supplementary Information

for

A Droplet Reactor on a Super-Hydrophobic Surface Allows Control and Characterization of Amyloid Fibril Growth

Table of Contents

Supplementary Note 1. Super-hydrophobic substrates design and fabrication............. 2
Supplementary Note 2. Super-hydrophobic substrates design and fabrication............. 3
Supplementary Note 3. Numerical model of convection in droplet and simulation......... 5
Supplementary Note 4. Samples preparation and characterization................................18
Supplementary Note 5. Lysozyme amyloid fibrils solution droplet on SHS...................21
Supplementary Note 6. 2D-XRD Measurements..22
Supplementary Note 7. Laser Confocal Raman Measurements......................................23
Supplementary Note 8. Fibrils formation from PHF6 24
Supplementary Note 9. Fibrils formation from Tau441 protein 26
Supplementary Note 10. SAXS and WAXS Measurements...27
Supplementary Note 11. Molecular dynamics (MD) calculation......................................31
References...44

Supplementary Note 1. Super-hydrophobic substrates design and fabrication

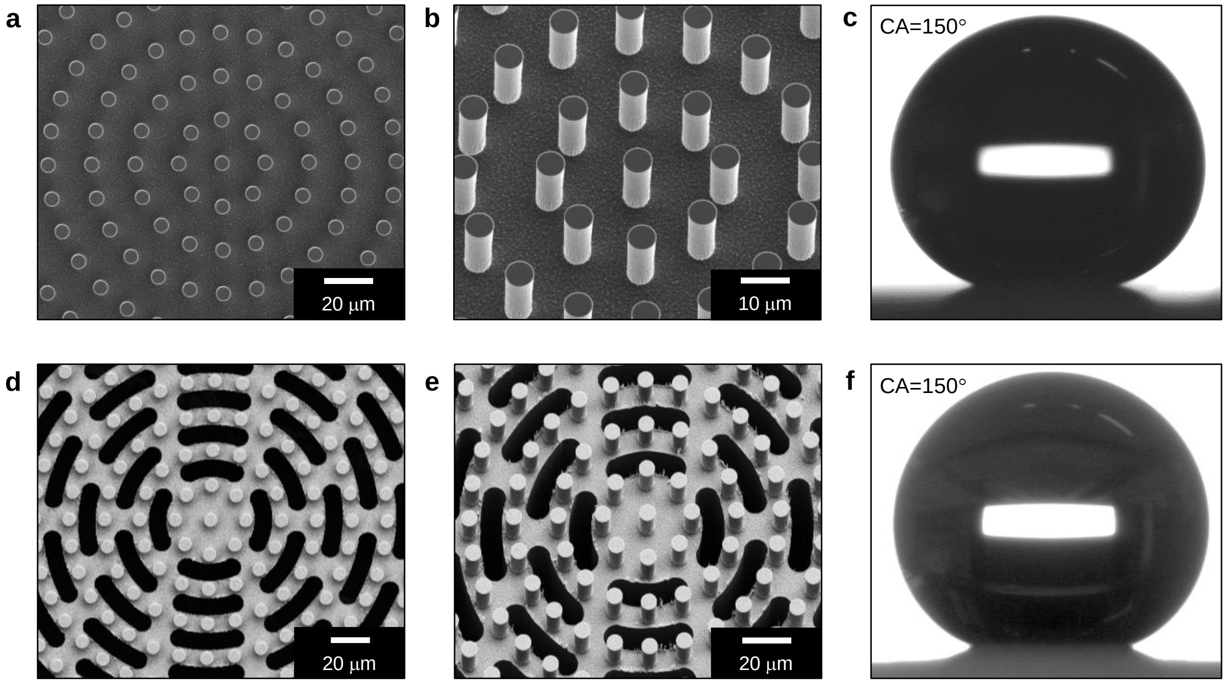

Supplementary Figure 1 SEM images and contact angle measurements of the manufactured SHS. **a**, SEM top view and **b**, 30° tilted view of SHS substrate with only pillars array. **c**, measured contact angle (CA) of SHS substrate with only pillars array. **d**, SEM top view and **e**, 30° tilted view of SHS substrate with pillars and holes array. **f**, measured CA of SHS substrate with pillars and holes array.

Supplementary Note 2. Super-hydrophobic substrates design and fabrication

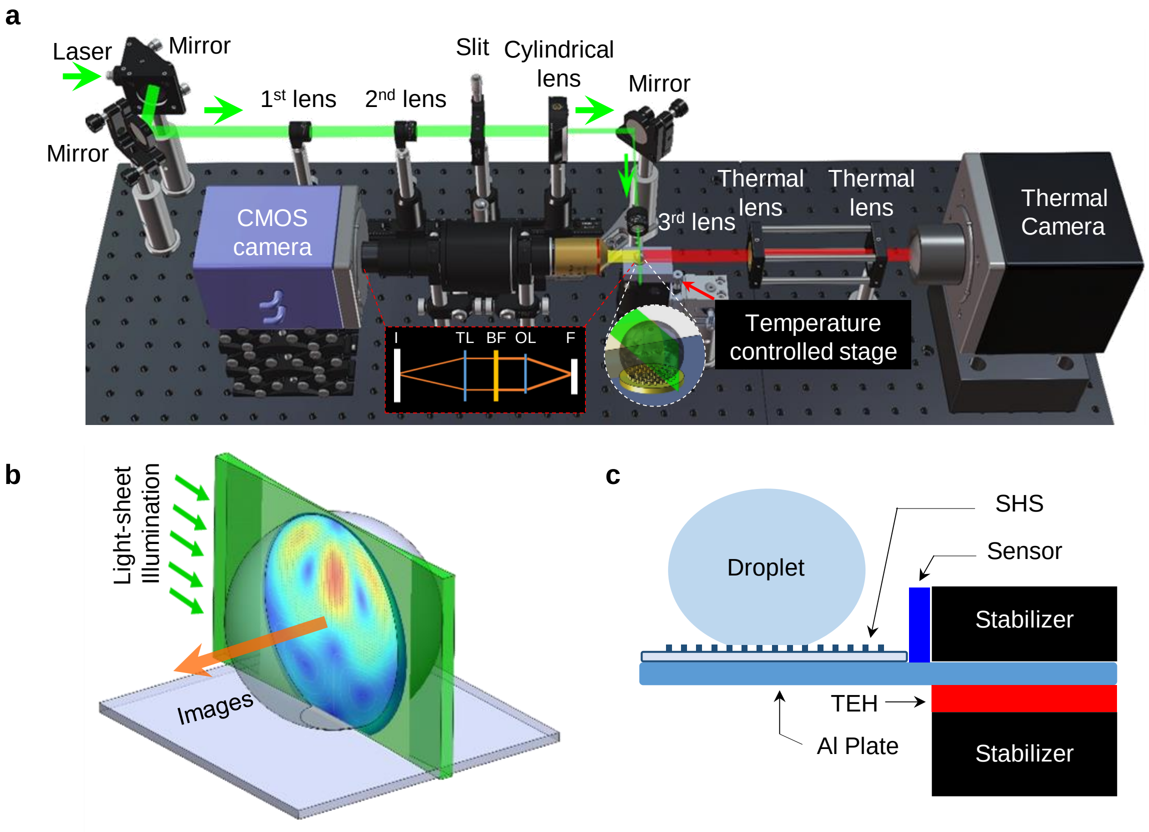

Supplementary Figure 2 Imaging system for convection characterization and temperature control system. **a**, Schematic diagram of the light-sheet based fluorescence microscopy imaging system. Insert sketch shows the images formation. **b,** light-sheet illumination model for a droplet; **c**, temperature control stage. SHS, super-hydrophobic substrate; TCS, temperature control system; THE, thermoelectric effect hotplate; F, fluorescence signal; OL, objective lens; BF, band-pass filter; TL, tube lens; I, images. The real experimental images are shown in **Figure 6 in main text.**

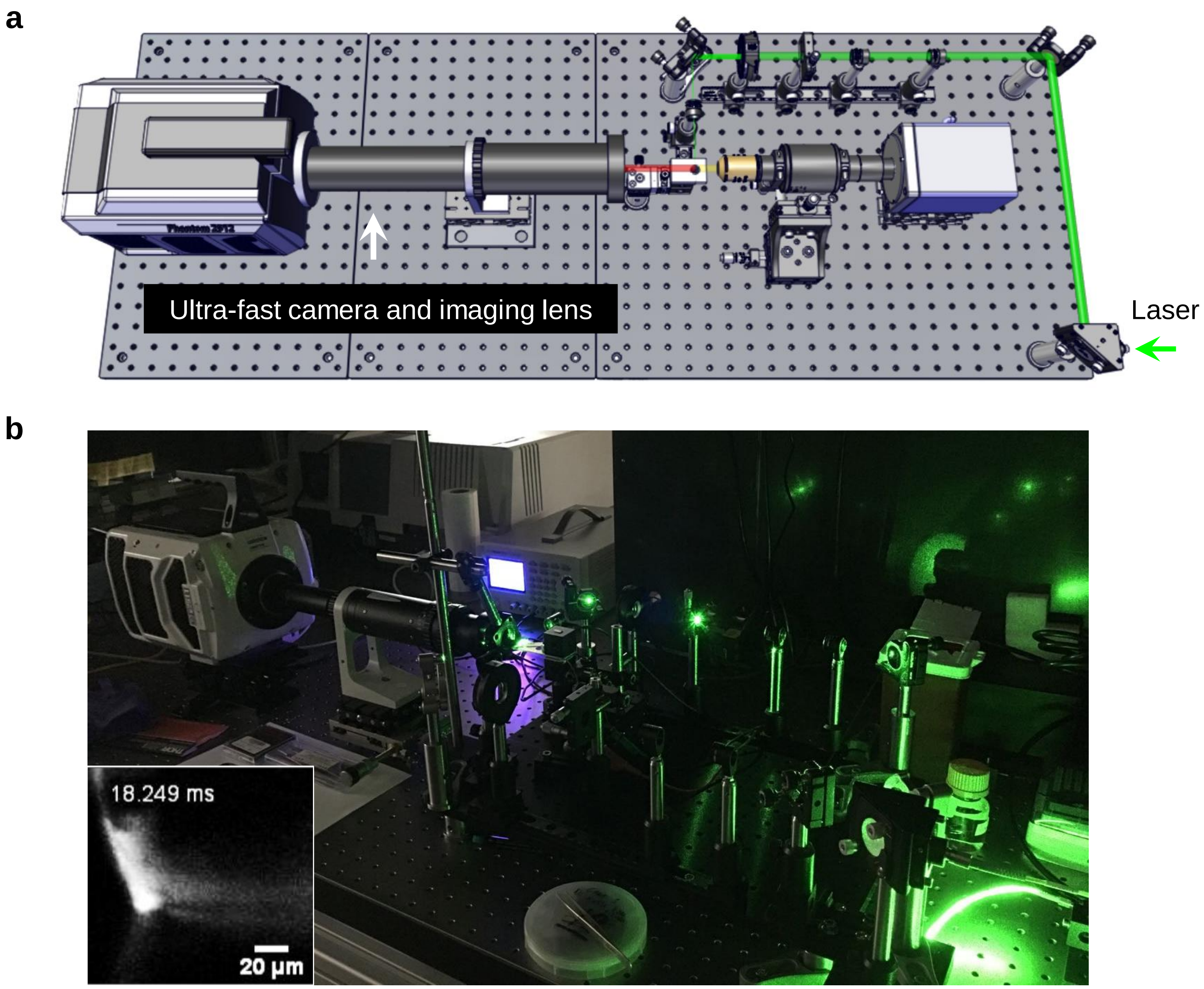

Supplementary Figure 3 Imaging system for droplet de-pinning dynamics. a, 3D model of the construction. **b**, real optical construction. Ultra-fast camera (Phantom v2511, Vision Research, USA) and Leica lens-set (2.0×9.2×1.6×). The insert picture in **b** shows the real optical images from the set-up.

Supplementary Note 3. Numerical model of convection in droplet and simulation

To study the flow field driven protein aggregation in real-time, a confined and steady flow is required. Although several mechanical stirring based systems had been reported, none of them showed the ability to do real-time investigations.[1-3] On the other hand, according to our previous research of droplet on SHS, the inherent convective flow in evaporating droplet should be a highly potential candidate as fiber aggregation driving force.[4-6] Here we produced a confined and steady convective flow driven by temperature gradient, ΔT (i.e., a water droplet with given size sitting on a heated substrate, $\Delta T = T_1 - T_0$, T_1 is the temperature of hot substrate, T_0 is ambient temperature).

For a evaporating droplet, its temperature distribution is complicated and difficult to control since it relies on both the contact angle and the ratio of substrate and liquid thermal conductivities.[7] However, consider a water droplet with given size sitting on a heated substrate, its temperature gradient can be expressed as following:

$$\Delta T = T_s - T_a \quad (1)$$

where T_s is the substrate temperature and T_a is ambient temperature.

At this condition, the temperature gradient will drive two convective flows with converse direction, capillary force based Marangoni convective flow and buoyancy force based natural convective flow.

The Marangoni convective flow and its intensity can be described as in Eq. 2:

$$M_a = \left| \frac{d\sigma}{dT} \right| \cdot \frac{a}{\mu\alpha} \cdot \Delta T \quad (2)$$

where M_a is the Marangoni number, σ is surface tension, T is temperature, ΔT is temperature gradient within the droplet, μ is kinematic viscosity of the liquid, α is the thermal diffusivity, and a is the diameter of the droplet.[8]

Meanwhile, natural convection driven by the buoyancy force is characterized with Grashof number (G_r), which is given by:

$$G_r = \frac{g\beta a^3}{\upsilon^2}\left(T_1 - T_2\right) = \frac{g\beta a^3}{\upsilon^2}\cdot\Delta T \qquad (3)$$

g is the gravitational acceleration, β is the thermal expansion coefficient, a is the characteristic length, υ is the kinematic viscosity, ΔT is temperature gradient within the droplet. And according to previous research, buoyancy force is negligible when $G_r < 2400$.[8]

In this research, for a water droplet with given volume (6.0 μL), the only factor affecting G_r is system temperature differences (ΔT) (β, *a and* υ are temperature independent in our gradient range) and it is represent as:

$$\begin{aligned} G_r &= \frac{g\beta a^3}{\upsilon^2}\left(T_1 - T_2\right) = \frac{g\beta a^3}{\upsilon^2}\cdot\Delta T \\ &= \frac{9.8\times 3.85\times 10^{-4}\times(2.25\times 10^{-3})^3}{\left(6.59\times 10^{-7}\right)^2}\cdot\Delta T = 99.55\cdot\Delta T \end{aligned} \qquad (4)$$

Therefore, for our specific system, when the ΔT < 24 K, the buoyancy effect is negligible.

The gradient ΔT could be controlled by a hot stage and measured directly with thermal camera (**Supplementary Figure 4**). Due to the heating transfer from hot stage to droplet, the temperature distribution in the droplet from bottom to the top is not linear at the initial state (**Supplementary Figure 4a**, the first 100 millisecond once droplet touched the hot surface), however, after 10 s, the heating transfer in droplet tend to steady state and the temperature distribution in droplet from bottom to top is close to linear (**Supplementary Figure 4b**). In addition, due to the heating transfer from droplet surface to ambient air, the measured ΔT in droplet is about 2 K lower than the setting point.

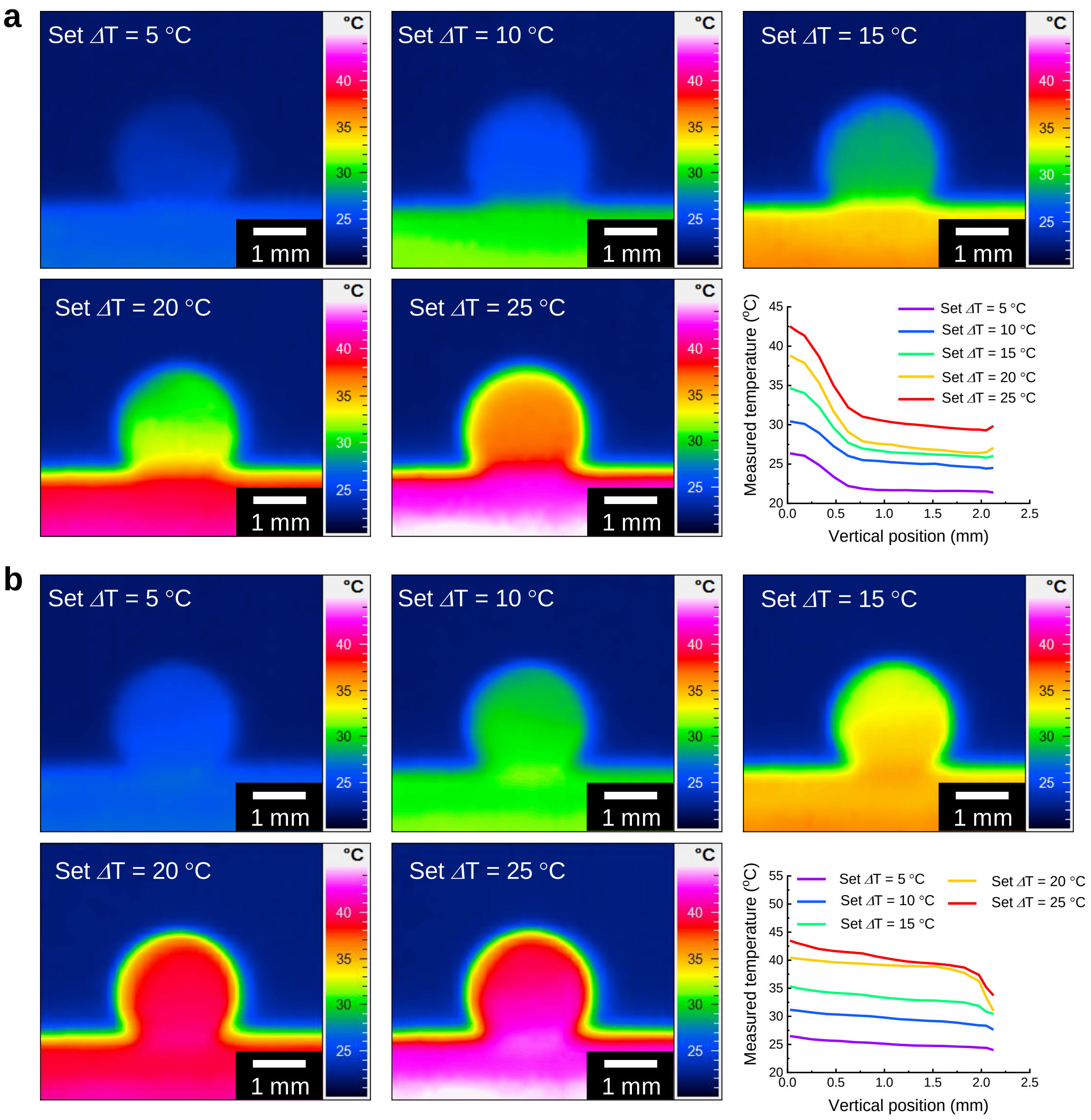

Supplementary Figure 4 Thermal images and temperature measurements in droplet on heated SHS. **a**, initial state and **b**, steady state after 10 s.

A 2D axisymmetric model was developed in ANSYS®, Release 19.1 (Fluent solver), which represents the fluid field and the heat transfer in a sessile droplet on a superhydrophobic surface that is heated in a stagnant environment. The model describes the internal dynamics of the drop with natural convection by buoyancy and Marangoni effect.[9,10] The schematic representation is

shown in **Supplementary Figure 5**. The continuity equation for the transient flow in each of the fluid zones (air and liquid) is:

$$\frac{\partial \rho}{\partial t} + \nabla \cdot (\rho V) = 0 \qquad (5)$$

In the Air phase, ideal gas law was used to estimate the density ρ and the momentum equation for solving the velocity field (V), laminar flow of Newtonian fluid can be written as:

$$\rho\left(\frac{\partial V}{\partial t} + V \cdot \Delta V\right) = \rho g - \nabla \rho + \nabla\left[\mu\left(\nabla V + (\nabla V)'\right) + \frac{2}{3}\mu(\nabla \cdot V)\right] \qquad (6)$$

For Liquid droplet, implementation of buoyancy-induced convection, the momentum equation can be written as:

$$\rho_l\left(\frac{\partial V}{\partial t} + V \cdot \Delta V\right) = \rho_l g - \nabla \rho + \nabla\left[\mu\left(\nabla V + (\nabla V)'\right)\right] \qquad (7)$$

Where the liquid viscosity μ is assumed constant and the density is a function of the temperature (T) using the Boussinesq approximation.

$$\rho_l = \rho_0\left[1 - \beta(T - T_0)\right] \qquad (8)$$

In the liquid, the energy balance to take into account the cooling by evaporation, and can be written as:

$$\rho C_p \frac{\partial T}{\partial t} + \rho C_p V \cdot \nabla T = \nabla \cdot (k \nabla T) - J\left[C_p(T - T_0) + L_v\right] \qquad (9)$$

Where C_p is a specific heat capacity, k is a conductivity of the liquid, in the equation (9) the last right side term is a heat released for evaporation, this is a function of latent heat of vaporization L_v and sensible heat $C_p(T\text{-}T_0)$ is a volumetric mass flow of water from the droplet to the air phase. For the Air phase the energy source term, was neglected. Since the time scale of volume change by evaporation is significantly larger than the thermal and fluid field time scale an assumption of quasi-steady state is acceptable

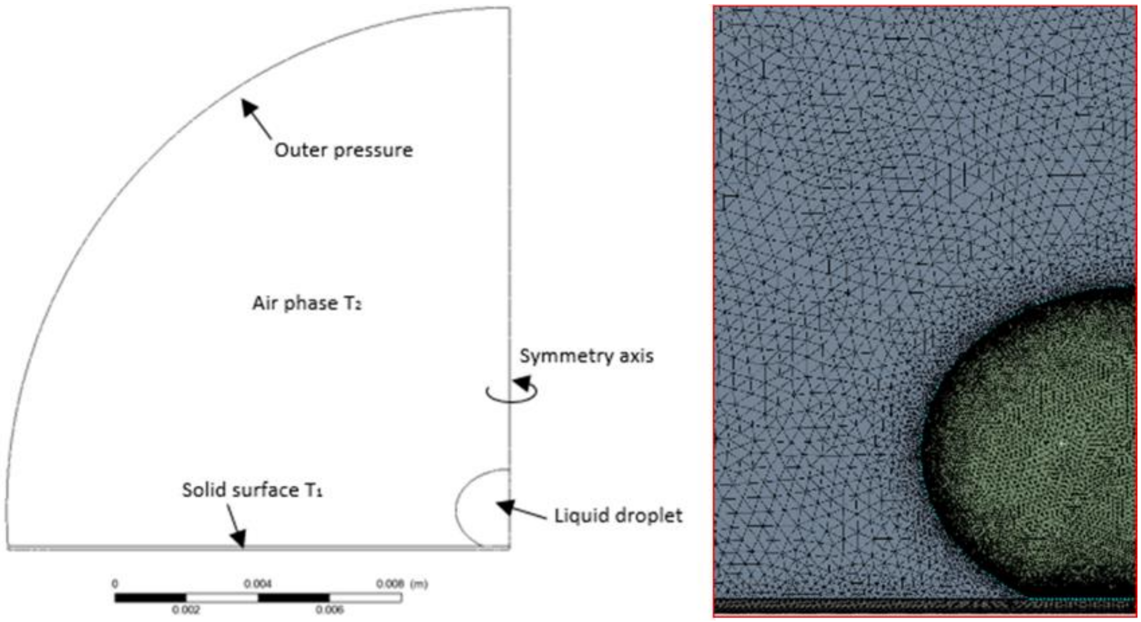

Supplementary Figure 5 Physical Domain and geometry mesh

In the Liquid-Air boundary conditions, two principals transport processes were taken into account, heat transfer by convection and stress boundary conditions by Marangoni effect. The Marangoni effect is used to describe the natural convection due to the changes in surface tension by temperature, on the other hand, the Solid-Liquid boundary condition was defined as a constant temperature, the same of the solid surface. The outer of the Air phase was defined as semi-spherical geometry with homogenous temperature and set as pressure outlet.

ANSYS®, Release 19.1 (Fluent solver) is used to solve the governing equations, using the pressure-based finite volume scheme, the pressure-velocity coupling is solved using the PISO algorithm with PRESTO spatial discretization for pressure and Second Order Upwind for Momentum, Density and Energy equations. The mesh is showed in detail in **Supplementary Figure 5**, in liquid phase ~33000 triangular elements, in solid phase ~15500 triangular elements, and ~39000 triangular elements in Air phase, the mesh cells in gas-liquid and gas-solid interphase are much finer than in the outer. The temperature difference between the solid surface (T_0) and the air (T_1) is called ΔT, and the details of the simulations are summarized in **Supplementary**

Table 1. In the temporary measure trace particles were added at the water to measure the internal convection, the increment of density for those particles is less than the 0.1%, for effects of the simulation the Boussinesq approximation to calculate the density reproduce the changes with an error in the same order of magnitude.

Supplementary Table 1 Fluid properties and simulation conditions

Delta Temperature	*[K]*	*25*	*20*	*15*	*10*	*5*
Time scale	**[s]**	10	12	15	25	34
Density(296.15K)	**[kg/m^3]**	997	997	997	997	997
Conductivity	**[W/(m K)]**	0.6	0.6	0.6	0.6	0.6
volume	**[m^3]**	6.53E-09	6.53E-09	6.53E-09	6.53E-09	6.53E-09
diameter	**[m]**	0.0023	0.0023	0.0023	0.0023	0.0023
7.1% of mass	**[kg]**	4.63E-07	4.63E-07	4.63E-07	4.63E-07	4.63E-07
Volumetric mass flow	**[kg/(m^{3}s)]**	7.09E+00	5.91E+00	4.73E+00	2.84E+00	2.09E+00
heat of evaporation	**[W/m^3]**	-17.554.396	-14.628.663	-11.702.930	-7.021.758	-5.163.058

At first, the convective flow was simulated with general Marangoni condition, i.e., surface tension is decreasing with increased temperature. The convective flow in droplet with three different given temperature gradients, ΔT = 5 K, ΔT = 10 K and ΔT = 15 K, were simulated (**Supplementary Figure 6**).

Experimentally, we studied and showed by optical imaging with the apparatus in **Supplementary Figure 2**, the convective flow field inside the droplet containing Rhodamine labeled melamine micro sphere (5.0 μm in diameter, ~500 beads per micro-liter), sitting on a hot-stage with particle tracking technique (**Supplementary Figure 6d-g** and **Supplementary Movie 1**). Initially we were expecting the convective flow driven by ΔT is steady and continues, as shown in simulation based on Marangoni theorem (**Supplementary Figure 6**). However, the experimental data clearly indicate the stream lines start from the bottom-edge of the droplet and end to the bottom-center, finally impacting the substrate and spreading away. Continuous convective flows are hardly to form. Although these convective flow fields in the droplets are strongly affected by the temperature gradient, i.e., ΔT, and the velocities increase with an

increasing ΔT from 0 K to 15 K in agreement with the Marangoni theorem, these profiles are not strictly symmetric due to the disturbance from crosswind airflow.[11] Hence, a continuous convective flow field which can drive the particles in droplet and without contact to the substrate is highly imperative.

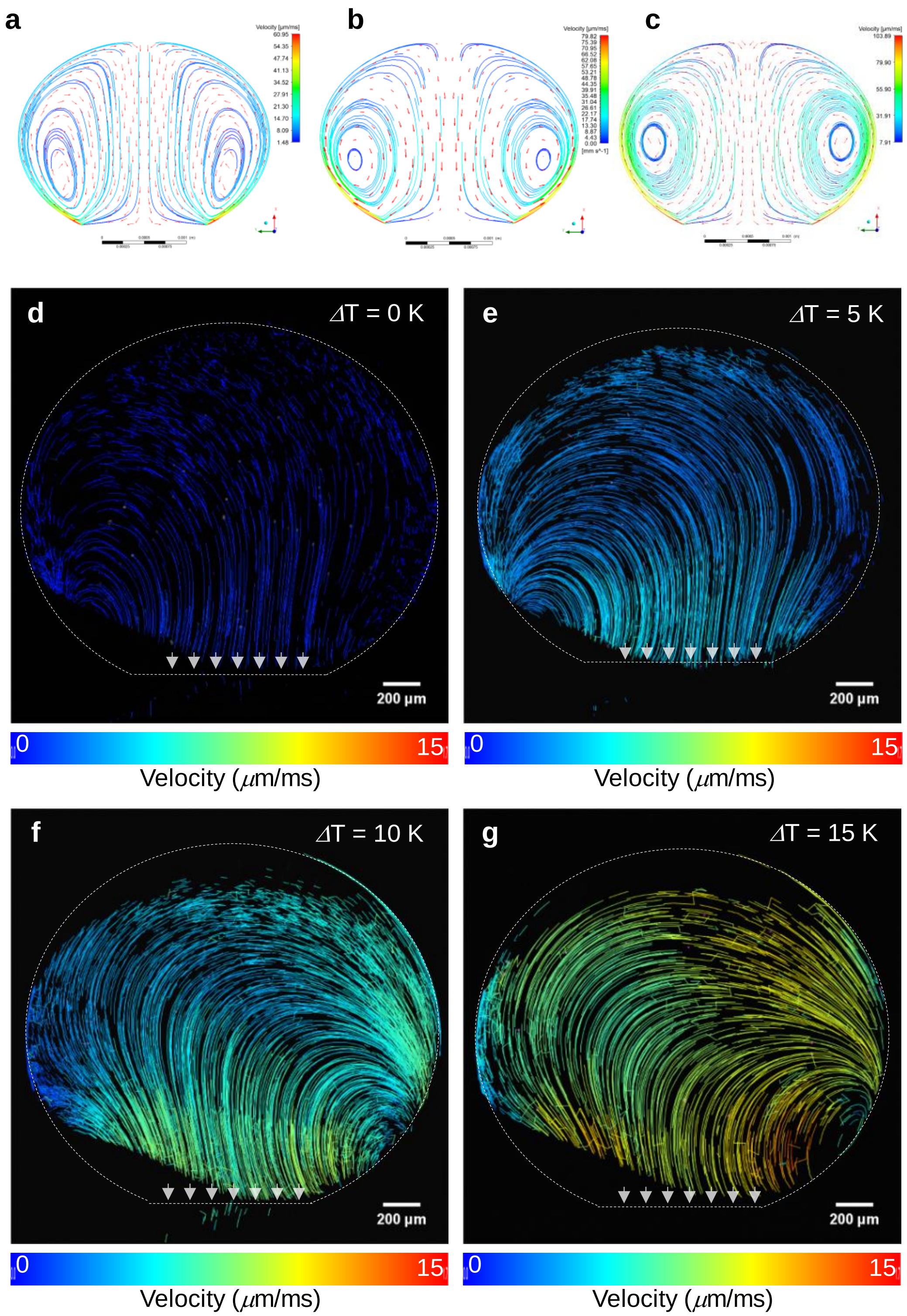

Supplementary Figure 6 Simulated and experimental convection flow in droplet on SHS with difference temperature gradients ΔT. a-c, simulations based on Marangoni effect with ΔT = 5 K, ΔT = 10 K and ΔT = 15 K. d-f, experimental convection flow in droplet with ΔT = 0 K, ΔT = 5 K, ΔT = 10 K and ΔT = 15 K.

Second, the convective flow was simulated with buoyancy effect. The convective flow in droplet with three different given temperature gradients, ΔT = 5 K, ΔT = 10 K and ΔT = 15 K, were simulated (**Supplementary Figure 7**). Close to the superhydrophobic substrate, the temperature is high, and the density goes down promoting the movement of the liquid, therefore, the buoyancy force drives liquid flowing away from the droplet bottom to the top and striking the substrate at the liquid-substrate contact edge. The continuous flow is absent due to the striking between liquid and solid substrate.

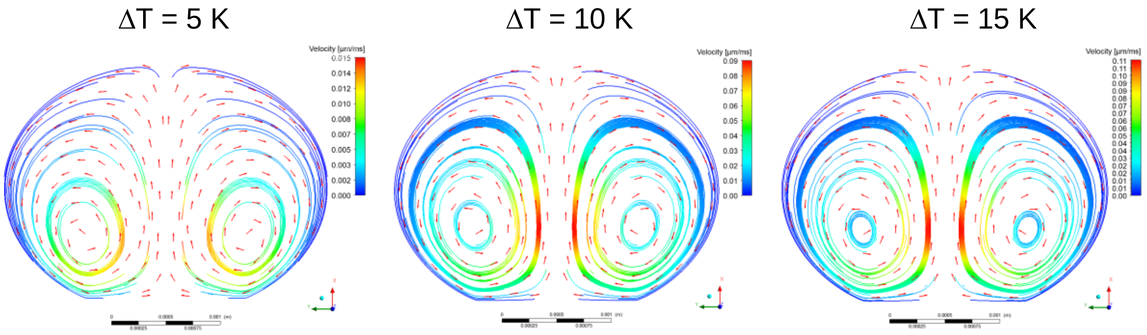

Supplementary Figure 7 Simulated convection flow in droplet on SHS with difference temperature gradients ΔT based on buoyancy effect.

However, with the temperature gradient increased to 20 K, two continuous convective flow formed at the top of the droplet (**Supplementary Figure 8** and **Supplementary Figure 9**). The results of the 2D symmetric simulation, velocity streamlines, temperature profile and strain rate magnitude of a sessile droplet with a ΔT = 20 K, are displayed in **Supplementary Figure 9**. In the streamlined right side symmetry profile, it is possible to observe two differentiated areas. The first one is the area at the bottom of the droplet, in this zone, the movement is in a clockwise direction, and is promoted mainly by the effect of the change of density for heating effect. The second area is at the top of the droplet is in a counterclockwise direction, and this is promoted mainly by the loss of heat by evaporation and convection with the surrounding gas.

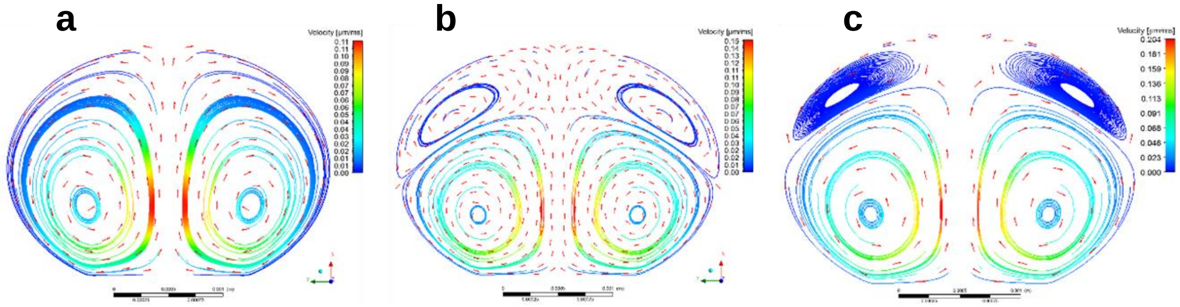

Supplementary Figure 8 Simulated convection flow in droplet on SHS with difference temperature gradients ΔT based on buoyancy effect. (a) ΔT = 15 K, (b) ΔT = 20 K, (c) ΔT = 25 K.

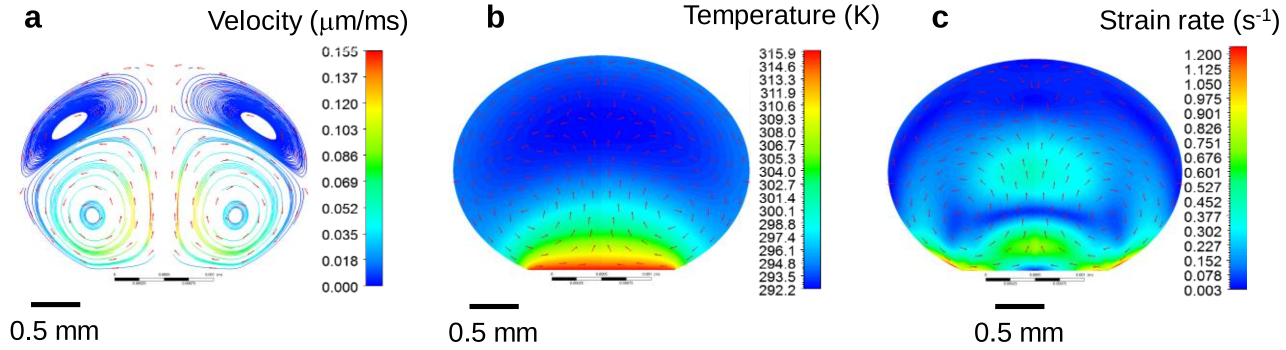

Supplementary Figure 9 Simulated convection flow in droplet on SHS with difference temperature gradients ΔT based on buoyancy effect Streamlines and velocity vectors (a), Temperature profile (b), Strain Rate magnitude (c).

The temperature profile of the droplet shows a monotonically decreasing of the temperature from the bottom to the top. Instantaneous conduction of heat from the substrate and the loss of energy by convection and evaporation in the surface, characterize the temperature profile. This temperature profile is the driving force for the internal convection due to buoyancy and the shear stress applied at the wall. The strain rate **Supplementary Figure 9c** shows a maximum corresponding to the stress with the triple point of contact (droplet, gas, solid). Another area of high strain rate is in just up of the substrate in the middle of the droplet, and this is because of two streams with a different direction of flow are converging in a field of high velocity.

Moreover, the difference in temperature ΔT plays a critical role in the behavior of the field of fluid, in **Supplementary Figure 8** is possible to observe that the increase of the difference of

temperature between the substrate and the surrounding air, reduce the magnitude of the up area in the droplet. It is reasonable due to the dissipation of energy is radial from the hot zone to the borders, and the flow movement is going in this direction. However, at low differences in temperature, the magnitude of the up area is increased.

In addition, the fluid field in real is affected by Buoyancy and Marangoni effect. To evaluate the effect that these parameters have in the internal fluid of the droplet, five study cases were carried out. In each one of them, a specific phenomenon was studied. So, Buoyancy, Buoyancy and Marangoni negative, Buoyancy and Marangoni positive, constant density and Marangoni negative, and the last case is constant density with Marangoni positive. The results for each of these cases, assessed in five ΔT cases are displayed in **Supplementary Table 2**.

Supplementary Table 2 Five case of flow flied driven by buoyancy effect and Marangoni effect in droplet.

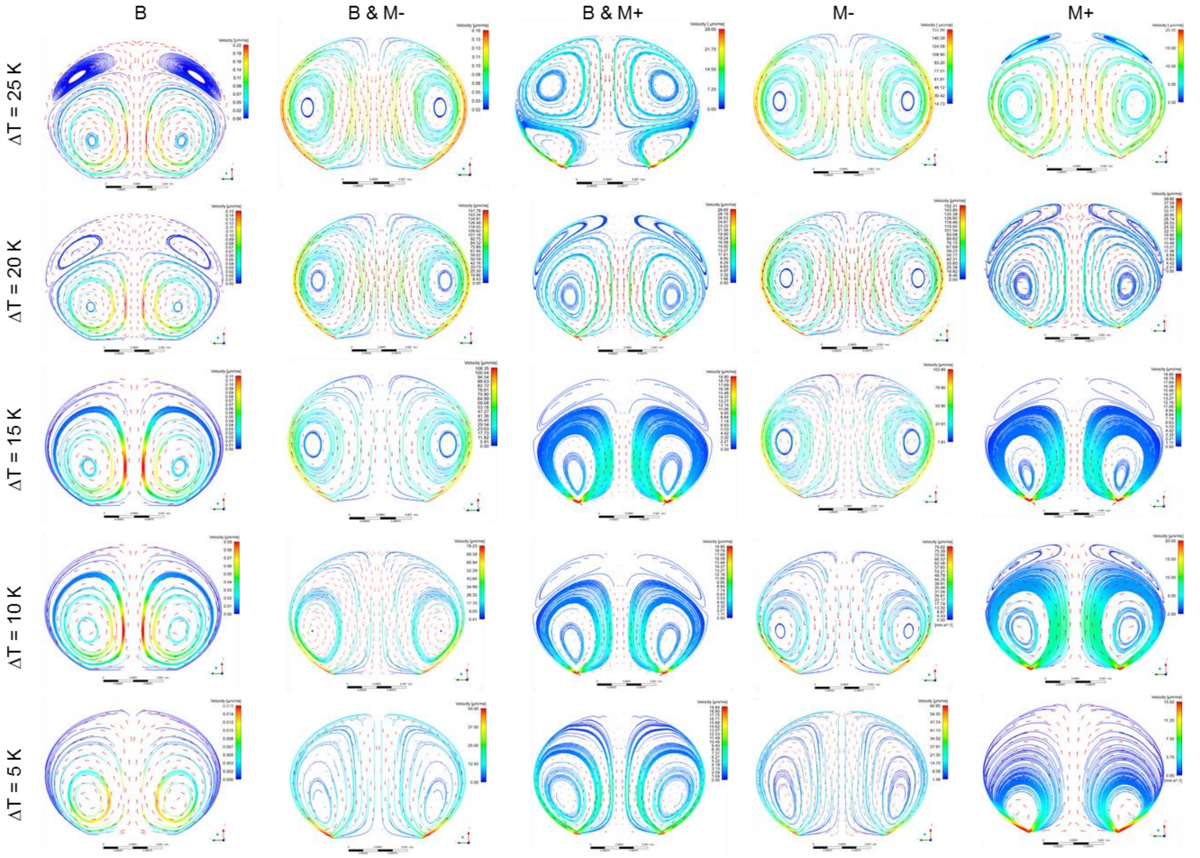

Pure Buoyancy condition (B): in this condition, the field of fluid is characterized by the movement of fluid from the center of the droplet to the borders and is promoted by the changes of density by the heating in the substrate. When the difference of temperature between the substrate and the surrounding air is high a significant thermal gradient is present, and in the top of the droplet a new vortex is formed.

Buoyancy condition and Marangoni negative (B & M-): the normal behavior of the water like a pure liquid shows a reduction of the surface tension with the increase of the temperature.[12] The rate of change of the surface tension promotes shear stress in the walls of the droplet; this means that the fluid travels close to the walls and return to the bottom by the middle of the droplet. With the reduction of the difference of the temperature of the substrate and the Air surrounding

Buoyancy condition and Marangoni positive (B & M+): Marangoni positive effect is possible in fluids with a self-rewetting fluids characteristics, or when particles add to the water increase rate of change of the surface tension with the temperature.[13] In this case, the strain rate close to the border is high than in the middle of the droplet. Then buoyancy effect allowed by the density change promotes the vortex in the top part, and the Marangoni promote the vortex formation in the bottom part of the droplet. With the decrease of the temperature, the surface temperature gradient is reduced, and the buoyancy effect loss effect and the Marangoni effect is dominant, thus produce a big vortex in all the droplet.

Constant density with Marangoni positive and Marangoni negative cases (M+ & M-): this simulation corresponds to the non-physical case because it is not possible to decouple these effects, and was made to study the magnitude of the Marangoni effect in the field of fluid, in both cases it is evident that the Marangoni effect preserves the fluid patterns inside the droplet and is mainly responsible for the vortex formation.

The deviation of the experimental results is the product of the non-symmetric behavior because the surrounding air is not in stagnation condition. The Marangoni effect was used to describe slip condition in the wall by the effect of the surface tension with the temperature. The internal behavior of the experiments with ΔT equal to 5 K, and 10 K does not show second recirculation area; due to the low surface temperature gradient, the variation of the surface tension is not significant, and the Marangoni effect is negligible. When the temperature gradient becomes higher than 10 K, the surface tension gradient in the air-water surface becomes more important. Therefore, the stress tensor at the wall is higher, and the velocity in the top area is lower than in the bottom, which allows the double recirculation.

Supplementary Note 4. Samples preparation and characterization

Lysozyme amyloid fibrils were produced starting from a solution of HEWL powder (Sigma) (10 mg/ml) in MilliQ water according to previous reports.[14,15] Acidification of the solution to pH 2.0 was obtained by adding 9% (v/v) of HCl (1N) in the solution. The mix was then immersed in a water bath at 60°C for 120 h until the formation of amyloid fibrils, as verified by AFM (**Supplementary Figure 10**). Briefly, the previously formed amyloid fibrils solution was diluted 5 times with MilliQ water of which a 30 μl drop was spotted on a freshly cleaved mica sheet. The sample was then rinsed several times and gently dried with an N_2 flux. AFM was then performed in JPK Nanowizard III by using a XSC-Al-BS probe (MikroMasch, GmbH), run in tapping mode at the resonance frequency of about 120 KHz. In addition, full length Tau (Tau441, 441 amino acids in length) was purchased in lyophilized form (buffer prior lyophilization: 50 mM Tris-HCl pH 7.5, 150 mM NaCl, 0.25 mM DTT, 0.1 mM PMSF) from SignalChem (Richmond, BC, Canada). PHF6 was purchased as acetyl-PHF6-amide-trifluoroacetate salt (sequence: VQIVYK) from Bachem (Bachem AG, Switzerland).

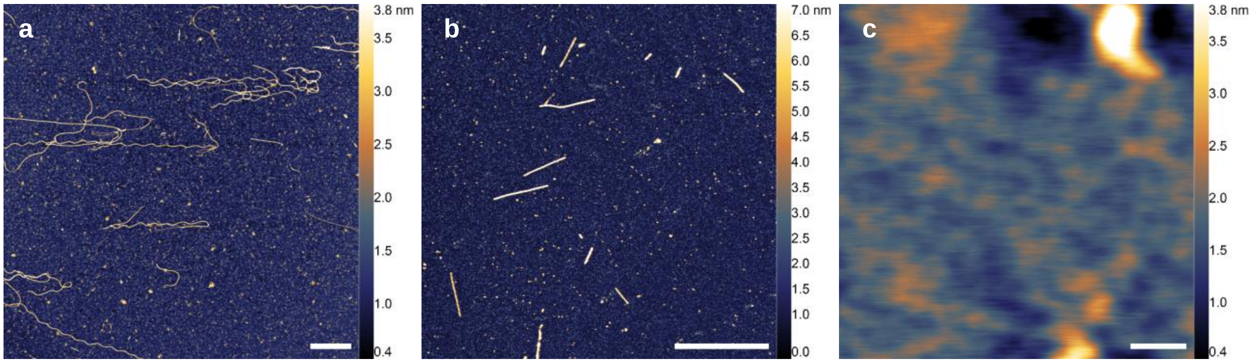

Supplementary Figure 10 AFM topography image of the HEWL, PHF6 and Tau441 samples. **a**, preformed amyloid fibrils from HEWL solution. **b**, Sample from PHF6 solution deposited before dropping on SHS **c**, Sample from Tau441 solution before deposition on SHS. Scale bars, 1 μm.

In **Supplementary Figure 10**, the AFM topography image of an area of about 20×20 μm is shown. The analysis of the typical lengths, heights and widths of the HEWL amyloid fibrils from

the AFM (**Supplementary Figure 10a**) gave the following results: width=18±3 nm, height=3.4±0.6 nm, length=19±7 μm. Notably, the width value of the actual fibril should be de-convoluted from the diameter of the tip to give results close to the real values. The fibrils have similar heights to those reported in the literatures.[15-17] Since the amyloid fibrils show a helical ultrastructure, it was also possible to measure their twisting pitch that evidenced mainly two populations of fibrils, one 76.5±1.2 nm long and the other 96.2±1.2 nm long (**Supplementary Figure 10a**).

Tau proteins are stabilizing proteins that act on the microtubules (MT) of the central nervous system and can be also found associated with microglia and astrocytes. In humans, six MAPT encoding genes are present, each having a different amino acid length. [18] Pathologic manifestation of Tau includes amyloid like deposits in the human brain. A long list of neurodegenerative diseases has been compiled that presents tau aggregates, among which Alzheimer disease (AD), Pick's disease, Chronic Traumatic Encephalopathy (CTE), Parkinson's disease, Progressive Supranuclear Palsy (PSP) are the most common (see[19] for a review). The onset of Tau pathologies has been associated both to Tau aggregation into amyloid form and to the spreading of its oligomers in a prion-like manner[19-24] and recently the interplay between Tau and amyloid-β, the other major component of amyloid plaques in the Alzheimer's brain, has been demonstrated to be quite complex[25]. Tau deposits in Alzheimer's patients (so called neurofibrillary tangles, NFT) are of amyloid nature and comprise paired helical filaments (PHFs) and straight filaments (SFs)[20]. Reports highlight the difficulty in providing models for full length Tau protein aggregation into amyloid fibrils. Its intrinsic disordered nature is a burden for classical methods of protein structure determination that, to be accurate, mostly rely on production of large ordered three-dimensional crystals of the proteins. Therefore often the target of study, the full length Tau is actually modified with recombinant fragments and does not include post-translational modifications[26]. Moreover, inducing fibrillation of full length Tau *in vitro*, needs the addition of a cofactors, such as e. g. heparin.[27] The most used techniques for the determination of intrinsically disordered protein (IDP) structure, are X-ray crystallography[28], NMR[28,29] and Small-angle

Scattering (SAS, both X-ray and neutron)[30]. However, despite the great progress of these techniques, the sample preparation and the accuracy of the results for structure determination are still a bottleneck. An available method to predict the portions of the molecule involved in the amyloid formation, is to test the aggregation propensity of short peptides by crystallographic methods. One of these synthetic peptides, the so called PHF6, with sequence ^{306}VQIVYK^{311} (PHF6 in R3 portion of Tau441, PDB file: 2ON9) is widely recognized to be involved in Tau aggregation[31-33]. In this work, both PHF6 and full length Tau441 were used to test our method of protein fiber and IDP formation and to provide an experimental benchmark of the relevant parameters that regulated the responsible physical phenomena.

In the case of PHF6 and Tau441, we wanted to verify if at time zero, right before the deposition on SHS, amyloid fibrils were already formed. In **Supplementary Figure 10b** the AFM topography performed on PHF6 sample right before deposition on the SHS shows several short fibrils of averaged a length value of 760±382 nm and a height of 4.69±0.82 nm. In the case of Tau441 (**Supplementary Figure 10c**) there were no amyloid fibrils pre-formed in the whole sample solution. This result is important for the discussion of the experimental part that follows.

Supplementary Note 5. Lysozyme amyloid fibrils solution droplet on SHS

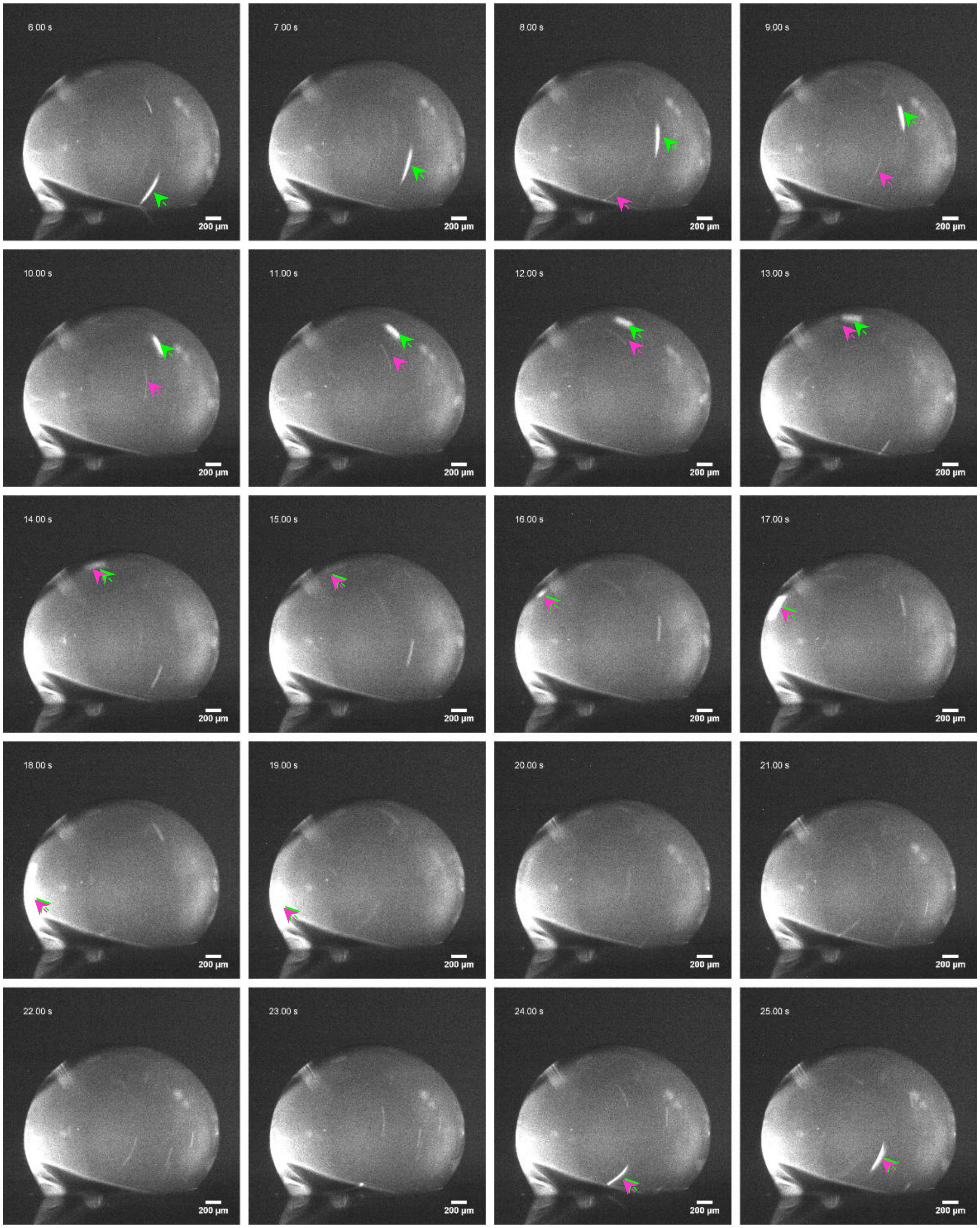

Supplementary Figure 11 Full frames tracking of amyloid fibrils driven by confined convection flow in droplet at ΔT = 25 K.

Supplementary Note 6. 2D-XRD Measurements

The SHS with micro-holes were used for protein self-aggregation and suspension from a sessile droplet. After drying at same condition, even the substrates alone (with no deposited proteins) were characterized by X-ray diffractometer with 2D detector (D8 Venture, Bruker, USA).To minimize the diffraction signal from substrate, the SHS was glued to stand on a tip vertically and orthogonal to X-ray source (Cu target, 50 kV) perpendicularly (**Supplementary Supplementary Figure 12**). The diffraction patterns were acquired by a detector at 40 mm distance with 600 s exposure time.

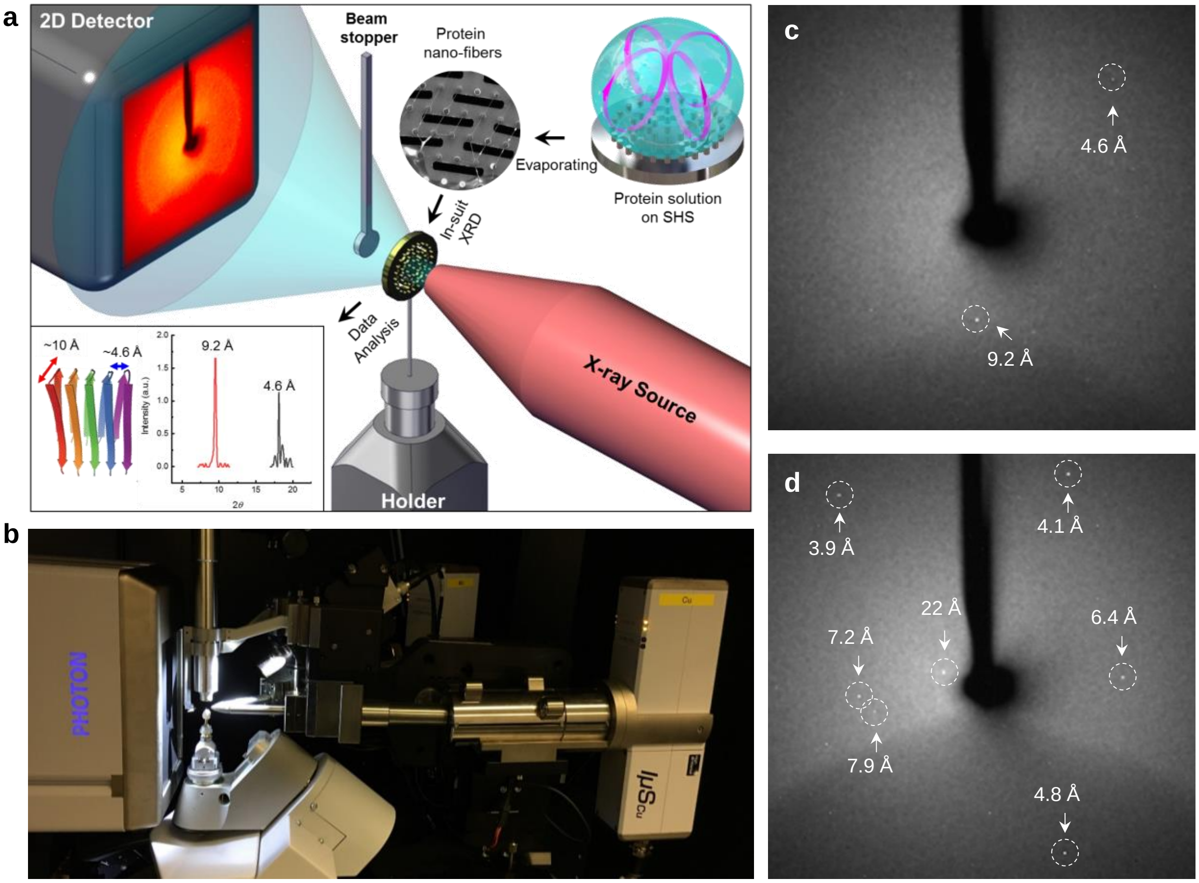

Supplementary Figure 12 In-situ 2D-XRD experimental set-up. **a**, schematic diagram and **b**, experimental set-up. **c**, 2D-XRD characterization of lysozyme amyloid fibrils suspended between two pillars. **d**, 2D-XRD characterization of hair-pin (HP) type lysozyme amyloid fibrils.

Supplementary Note 7. Laser Confocal Raman Measurements

Raman spectrum of the deposited protein fibrils were measured with a laser confocal Raman microscopy (Alpha300 RA, WITec, Germany). The back Raman scattering signal under 4-mW 532 nm laser (Coherent Compass Sapphire Laser) was collected by a 100× objective lens (Zeiss, EC EPIPLAN NEOFLUAR, 0.9 NA) and then detected by a CCD detector (DU970) at -65°C. For each measurement at least three spectra were collected, baseline subtracted with a polynomial curve of grade 5, normalized at the peak 1450 cm^{-1}, and averaged. Fitting of Amide I peak was performed by using three Voigt functions each centered at 1655, 1668 and 1685 cm^{-1}, related to α-helix, β-sheet and disordered secondary structure of the protein, respectively.

Supplementary Note 8. Fibrils formation from PHF6

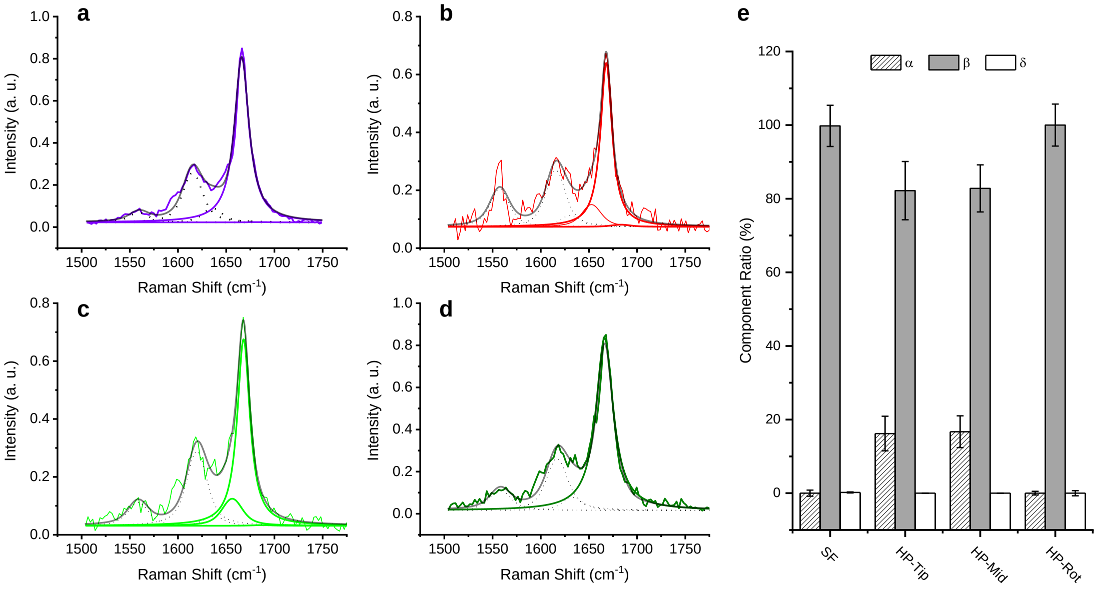

Supplementary Figure 13 Raman spectra of thePHF6 suspended fiber and of PHF6 hair-pins performed at different hairpin positions. a, Raman measurements performed in the middle of a suspended fiber. **b**, Raman measurement performed on the tip of the hairpin. **c**, Raman measurement performed in the middle of the hairpin and **d**, Raman measurement performed at the hairpin bottom. **e**, histogram plot of component ratios (%) of the secondary structures components analyzed in the different conditions.

Due to our final interest in Tau protein structure we analyzed in our study the fiber structure of PHF6 and Tau441 after formation on our SHS samples. As in the lysozyme case, we performed the PHF6 Raman measurements at different heights along the protein hairpin and analyzed their composition in terms of secondary structure (**Supplementary Figure 14**). In **Supplementary Figure 14**, the plots show the experimental spectra and the fittings of the three components of the Amide I in the Raman measurement, peaked at about 1655 cm^{-1}, 1668 cm^{-1} and 1685 cm^{-1}, representing respectively α-helix, β-sheet and disordered structures. Hair-pin tip, middle and bottom spectra (**Supplementary Figure 14b, c, d** respectively) are compared with the Raman measurement on the suspended fiber (**Supplementary Figure 14a**) and resumed in the histogram plot of percentage ratio in **Supplementary Figure 15e**. Both the suspended fiber and the bottom of the hairpin show an almost 100% composition of β-sheet secondary structure. A slight difference appears in the case of the tip in the middle of the hairpin, where almost 20% of α-helix appears. In all these cases the presence of disordered structure detected was not significant. If we compare these results with the ones on the Lysozyme hair-pins, we can notice that PHF6 appears to have a major component of β-sheet structure even at extreme conditions (HP-tip). This can be explained by noticing that PHF6 is a short peptide and therefore is predictable that its structure will maintain a certain degree of ordered structure even if snapped-off by the breaking of the capillary during drop evaporation.

Supplementary Note 9. Fibrils formation from Tau441 protein

In the Raman spectra, a strong peak at 1670 cm^{-1} is evidencing the β-sheet structure formation in the suspended fibrils, the data is shown in **main text Figure 7.**

Supplementary Note 10. Small Angle X-ray Scattering (SAXS) and Wide Angle X-ray Scattering (WAXS) Measurements

Small Angle X-ray Scattering (SAXS) and Wide Angle X-ray Scattering (WAXS) data were collected at the X-ray MicroImaging Laboratory (XMI-Lab) which is equipped with a super bright synchrotron class table top X-ray micro-source (Cu Ka, λ = 0.15405 nm, 2475W, rotating anode) coupled, by a multilayer focusing optics (Confocal Max-Flux; CMF 15-105), to a SAXS/WAXS three pinhole Rigaku SMAX-3000 camera[36] (**Supplementary Figure 14a**). Two distinct detectors have been used for data collection: a Triton 20 gas-filled proportional counter (1024x1024 array, 195 μm pixel size) for SAXS, at about 2.2 m sample-to-detector distance (SDD), calibrated by using an Ag behenate powder as reference material; an image plate (IP) detector with 100 µm pixel size for WAXS, at a 28 mm SDD, calibrated by using a Si standard (NIST).[34,35] Samples were mounted in transmission mode onto a holder with holes (**Supplementary Figure 14b**) and measured in vacuum (~5 x10^{-2} mbar). The 5.5 mm beam stopper, before the SAXS detector, contains a pin diode to collect the beam transmitted through the sample. The beam is focused down to a size of about 200 μm to scan across a ~4x4 mm^2 sample area, collecting per each sample position 2D SAXS and corresponding transmitted intensity value. At the end of the serial collection scheme, several microscopic images (**Supplementary Figure 14c-e**) are composed by using a software, SUNBIM, developed at the XMI-Lab.[36]

SAXS and WAXS data were collected for samples Lysozymes and Tau441 in transmission geometry in order to underline the fiber nature of the β-sheet fibrils suspended on SHS.

For each sample, three microscopes have been extracted from raw SAXS data, here reported in **Supplementary Figure 14c-e** for Tau441: the transmission coefficient (**Supplementary Figure 14c**); the SAXS intensity scattered in the entire q-range (0.01-0.2Å^{-1}), shown in **Supplementary Figure 14d**; the ratio between the previous two microscopies (SAXS scattering normalized to transmission coefficient), shown in **Supplementary Figure 16e**.

Subsequently, 2D WAXS data have been collected in precise sample positions chosen in **Supplementary Figure 16e**. Precisely, three regions have been explored for Tau441: a single sample position, marked as *1* in **Supplementary Figure 16e***,* which corresponds to the highest normalized scattering and is clearly spatially related to the agglomeration area in **Supplementary Figure 16b**; a second single sample position, marked as *2* in **Supplementary Figure 14e**, which corresponds to a region with a uniform medium scattering (colored in cyan in **Supplementary Figure 14e**) and an internal 1.4×0.4 mm^2 area marked with the white rectangle (*3*). The corresponding 2D WAXS data, reported in **Supplementary Figure 14f (*1*), Supplementary Figure 14g (*2*)** and **Supplementary Figure 14h (*3*)** respectively, display a quite different intensity distribution. Precisely, **Supplementary Figure 14f** shows a mixture of rings and arcs, as expected for the agglomerated sample region in *2*, which contains different contributions (ordered and disordered aggregated fibrils, plus other residues); **Supplementary Figure 14e** is characterized by arcs only, as the typical fiber diffraction pattern of well aligned fibrils; **Supplementary Figure 14h** is made by full rings as expected in the case of a larger area with fibrils oriented in several directions. The agglomerated area is not interesting in this work; therefore **Supplementary Figure 14f** was not further analyzed.

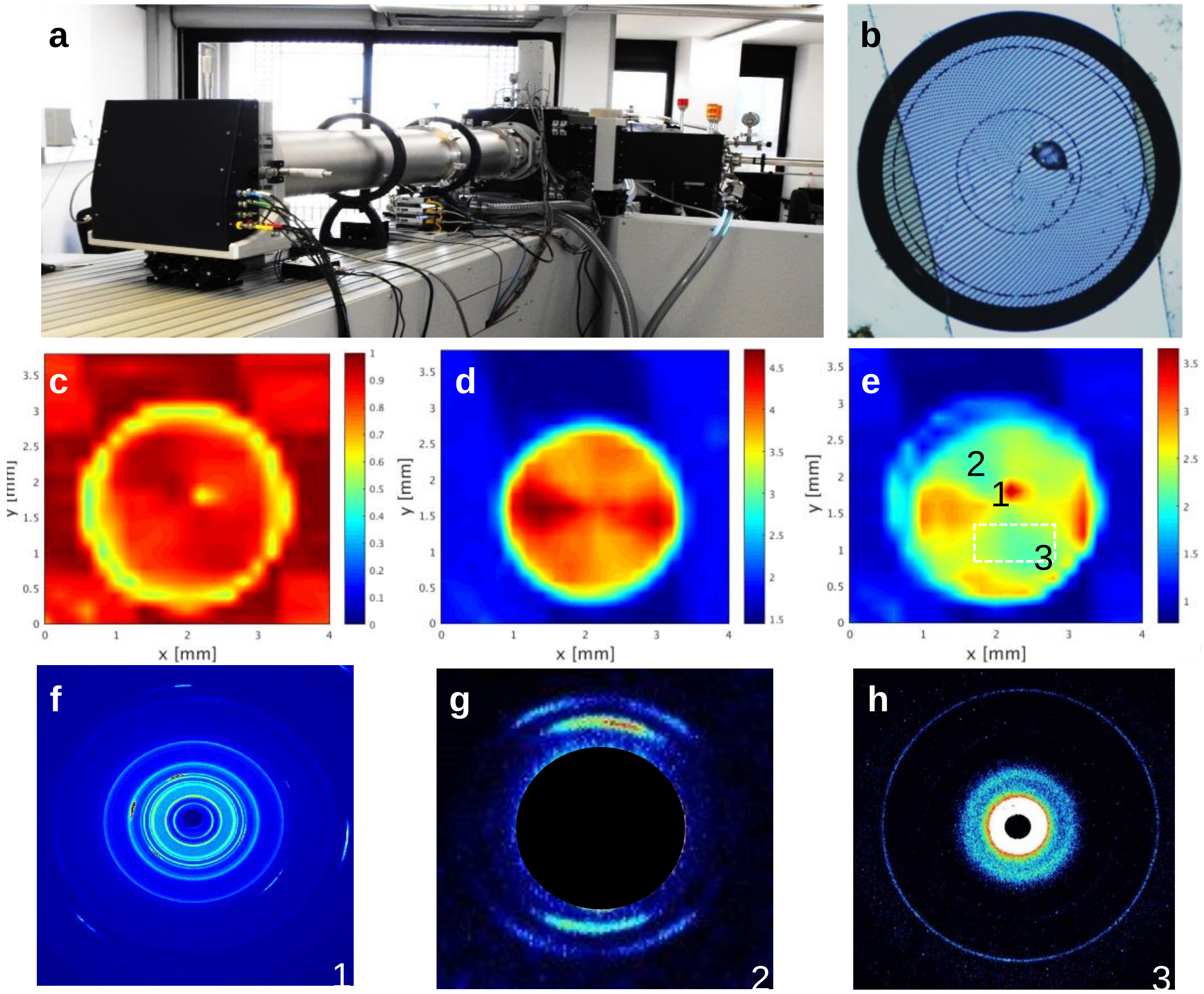

i	Sample	Peak label	q (Å^{-1})	D(Å) ± 0.5
		1	0.51	12.3
		2/2'	0.64/0.75	8.3/9.8
	Tau441	3	0.82	7.7
		4	1.00	6.3
		5	1.07	5.8

Supplementary Figure 14. **SAXS and WAXS measurements of Tau441 samples**. **a**, experimental facility. **b**, protein fibrils suspend on SHS with micro-pillars and holes array. **c**, mapped transmission coefficient. **d**, SAXS intensity scattered in the entire q-range (0.01-0.2Å^{-1}). **e**, the ratio between the previous two microscopies (SAXS scattering normalized to transmission coefficient). **f-h**, 2D WAXS data corresponding to 1, 2, and 3 in **e**. **i,** the peaks and corresponding distanced, measured for Tau441.

We focused on **Supplementary Figure 14g** and **Supplementary Figure 14h** which were reported in **Main text Figure 7d** and **Main text Figure 7g**, respectively. After centering, calibration, 2D-1D folding, and background subtraction, the final 1D WAXS profiles were extracted and displayed in **Main text Figure 7e** and **Main text Figure 7h**, respectively. **Main text Figure 7e** shows two major peaks, marked as “1” and “2”, plus other faint additional peaks. These two major peaks were fitted by Gaussian function (**Main text Figure 7f**) to determine the peak position of 0.51 and 0.64 $Å^{-1}$, which means that the lattice planes distances are of 12.3±0.5 (“1”) and 9.8±0.5 Å (“2”), respectively. The same procedure of data reduction was repeated also for **Main text Figure 7g**, resulting in the profile in **Main text Figure 7h**. Here, apart for peak “2”, which is slightly moved with respect to the same peak marked as “2’” in **Main text Figure 7e**, additional peaks were measured. The fit of the most intense of all, marked as peak “5”, is reported in **Main text Figure 7i**. The peaks and corresponding distances measured for Tau441 are shown in **Supplementary Figure 14i**.

Therefore, with the conjoint analysis with Raman, XRD and WAXS, we can confirm that these suspended fibrils are highly aligned β-sheet amyloid fibrils. In addition, we suggest that these fibrils have long range anisotropic order along the fiber axis.

Supplementary Note 11. Molecular dynamics (MD) calculation

Supplementary Note 11.1. Molecular dynamics protocol

Molecular dynamics simulation is a powerful method to understand proteins providing an atomistic view of molecular interactions and dynamics.[37-42] We applied molecular dynamics (MD) in explicit waters on PHF6 and Tau441 fibril models in order to have detailed insights of the fibril structural properties. Our trajectory analyses corroborate the stability profiles, experimentally observed, of the fibrils.

Molecular dynamics simulations of PHF6 (50ns) and Tau441 (two morphologies for 30ns each) were performed applying the same protocol. In details, the systems were solvated in an octahedron box using the TIP3P water models[43] with a 1.2 nm distance to the border of the molecule using GROMACS (5.1.2) and parametrized with Charm27 force field.[44,45] Electrostatic interactions were treated using the Particle Mesh Ewald method with a non-bonded cutoff of 1.0 nm for the Lennard-Jones potential under NPT conditions (298 K and 1 bar). The Berendsen algorithm[41] was applied to retain temperature and pressure, with a coupling constant of 1 ps for both parameters. All bond lengths were constrained with LINCS algorithm[42] and periodic boundary conditions were employed. Two femtoseconds (fs) time step was used to integrate the equation of motion. The water molecules were relaxed by energy minimization and followed by 10 ps of MD at 298 K, restraining the atomic positions with a harmonic potential. The systems were heated up gradually to 298 K in a six-step process (50 K to 298 K). Finally, a production runs were performed under NPT standard conditions, without restraints, for 30 ns. The analyses of the MD trajectories were done using the GROMACS (5.1.2) and Pymol packages (http://www.pymol.org).[46]

Supplementary Note 11.2. PHF6 fibril model description

A PHF6 fibril model was created starting from the PHF6 oligomer state solved by Sawaya, *et al*. with X-ray diffraction.[32] In details, several consecutive modules of the 2ON9 pdb coordinates

were firstly acetylated at the N-terminus and then replicated along both longitudinal and orthogonal fibril axes, adapting a previous modelling protocol.[47,48] **Supplementary Figure 15.** The obtained model represents a growth/elongated PHF6 fibril state consisting of 144 peptide units (12 layers of 6 dimers, perpendicular to the fibril axis, **Supplementary Figure 15**). To better mimic the systems observed at Raman spectra the MD simulation covered 50 ns.

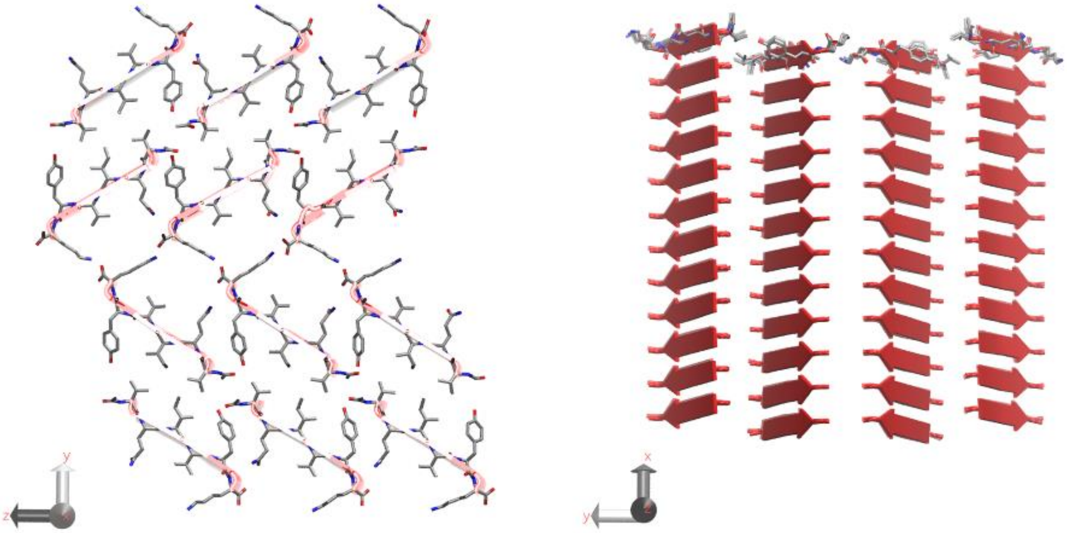

Supplementary Figure 15 PHF6 fibril architecture. Top (left, in direction to the fibril x axis) and frontal (perpendicular to the fibril axis, x) view of the PHF6 fiber initial model. Twelve copies of 6 PHF6 dimer peptides forming a fibril. Only the peptide heavy atoms of the first layer are displayed in sticks and colored by type (Oxygen: red, Nitrogen: blue, Carbon: white). Red arrows represent the β secondary structure.

Supplementary Note 11.3. Molecular Dynamic simulations of PHF6 aggregates

Molecular dynamics (MD) simulations in explicit water were carried on PHF6 fibril models to better understand the structure and dynamic properties of PHF6 aggregates. which represents the aggregation prone fragment of the Tau441 protein an important model in our study, because it is a building block to understand the structure of the more complex case of Tau441. We created a PHF6 fibril composed by 144 peptides in pre-defined β-sheet conformation using the coordinates of the pdb X-ray structure which shows highly ordered cross β PHF6 architecture (method section for details, **Supplementary Figure 15**). The obtained model represents an

elongated/a grown fibril state of PHF6 oligomer solved by Sawaya which is the repeat unit of PHF6 aggregates,[33] it contains 12 β-sheets peptide layers parallel to the fibril axis (**Supplementary Figure 15,** x axis), each composed by 12 peptides in strand extended conformations perpendicularly to the fibril axis (**Supplementary Figure 15**, x axis)

As shown in **Supplementary Figure 16a**, after 50 ns of MD simulation the PHF6 fibril moderately twists along the fibril axis yet retaining the overall β-sheets arrangement and without disassembly, as already seen in previous studies.[49]

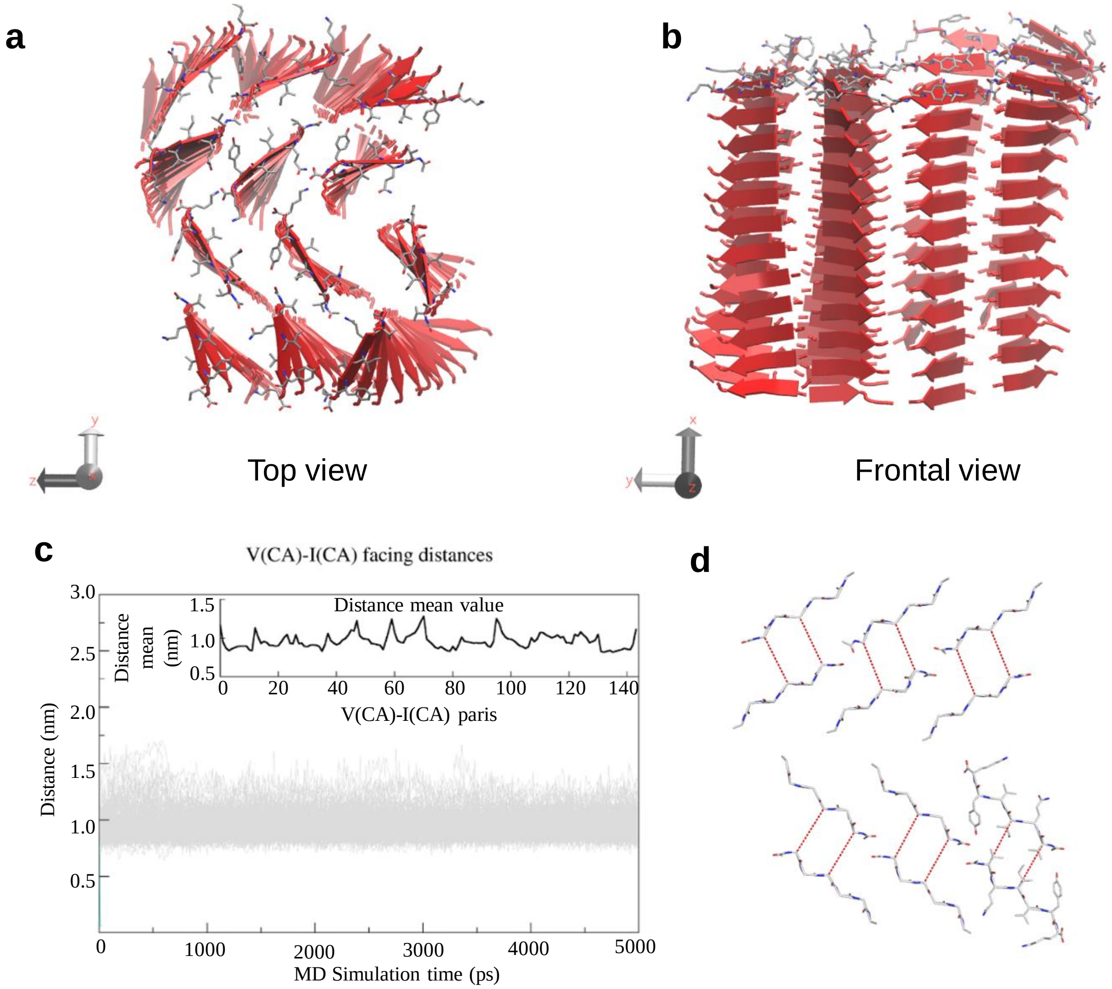

Supplementary Figure 16 MD simulation the PHF6 fibril after 50 ns. **a**, top view and **b**, frontal of the ending frame of PHF6 Molecular Dynamics. Only the peptide heavy atoms of the first sheet are displayed in sticks and colored by type (oxygen: red, Nitrogen: blue, carbon: with). Red arrows represent the βsecondary structure. **c**, **PHF6 MD2bis**, center of mass distances of the C_α atoms of each Valine residue versus the facing Isoleucine within each dimer along the PHF6 simulation. The top panel report the mean values of all the 144 distances averaged along the simulation. **d**,

six PHF6 dimer peptides forming a fibril. For clarity, the peptide backbone heavy atoms of the first layer are displayed in sticks and colored by type (oxygen:red, Nitrogen: blue, carbon: with), in one PHF6 dimer all heavy atoms are displayed to show the face-to-face arrangement of steric zipper arrangement. Red dashes represent the distances between the C_α atoms of facing Valine and Isoleucine residues

The Cα-root mean square deviation (RMSD) profiles respect to the starting model suggest that the overall system is stable till the end of the trajectory, with a slightly higher level of rearrangements affecting the terminal regions (layers 1-2 and 11-12). Indeed, the core fibril (layers 3 to 10) show RMSD mean values spanning from 0.24 to 0.31 nm, but those of the first and the last layers reach mean values of 0.45 and 0.46, respectively (**Supplementary Table 4** and **Supplementary Figure 17**). The secondary structure components result consistent with the initial model (**Supplementary Table 5** and **Supplementary Figure 18**) among the whole simulation. The 50% of residues show a β and the 33% a coil structure. The inter-layer distances between the Cα atoms of each layer versus its successive are extremely constant, mirroring a remarkable compactness of the overall system (**Supplementary Table 6**).

Notably, the distances between the C alpha atoms of Valine and Isoleucine facing residues within the fibril and conferring the typical face-to-face arrangement of class 1 steric zipper, as defined by Sawaya, are extremely conserved along the simulation (**Supplementary Figure 16b**) spanning from 8.2 to 12.8 Å, with narrow standard deviations (0.2 Å to 1.8 Å) among the 144 distances composing our model. This distance explains the pick values of PHF6 at 9 Å, registered at higher temperature.

Even though no conformational transition occurs during the calculations, the higher rearrangements of the terminal layers remark a different behavior of the terminal fibril portions, consistently with the spectroscopic measurements at the PHF6 hair-pin region. The absence of a complete helix transitions registered with Raman spectra along the trajectory may be likely hampered by the limited time scale applied and would require further theoretical investigations.

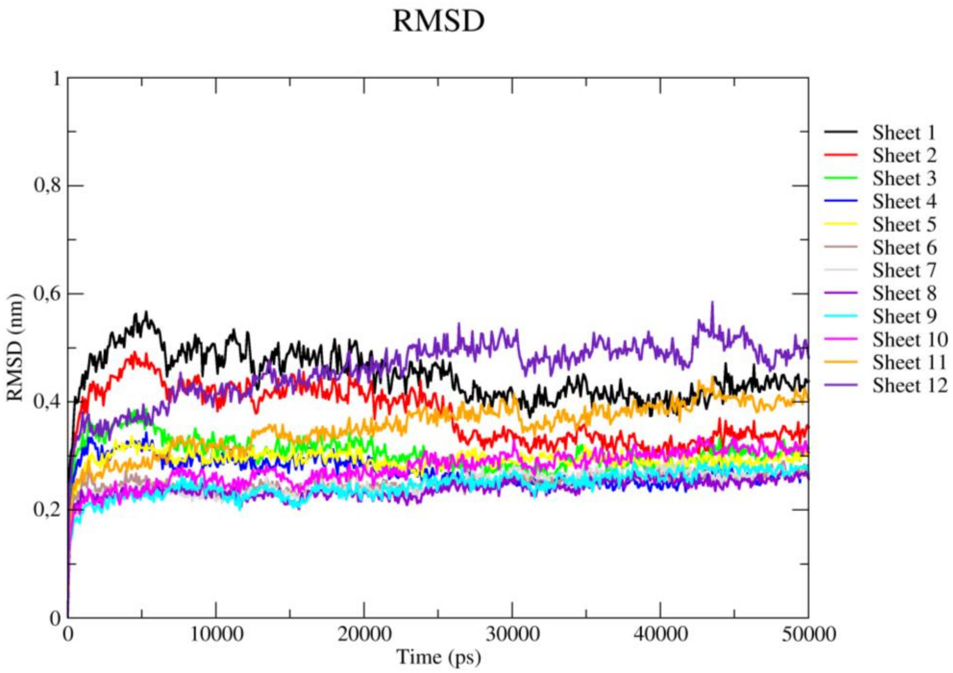

Supplementary Figure 17 Root mean square deviation (RMSD) profiles computed for each fibril layers along the MD simulation.

Supplementary Table 4. Root mean square deviation (RMSD, nm) mean and standard deviation values among the PHF6 MD simulation

Layer	Mean	SD
1	0.45	0.05
2	0.38	0.05
3	0.31	0.03
4	0.27	0.03
5	0.29	0.02
6	0.25	0.02
7	0.25	0.02
8	0.24	0.02
9	0.25	0.03
10	0.28	0.03
11	0.35	0.05
12	0.46	0.06

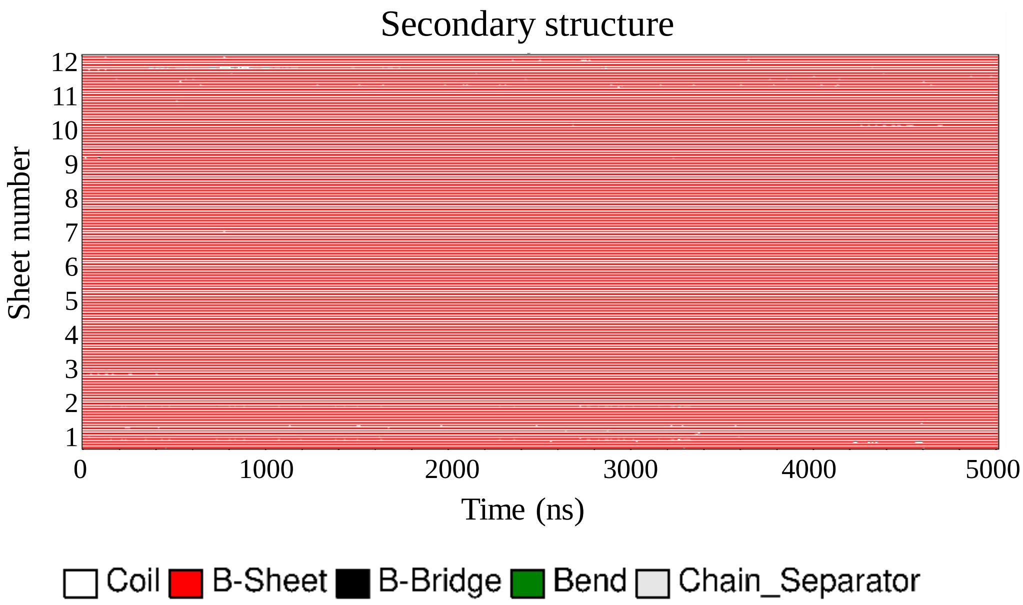

Supplementary Figure 18 Secondary structure components computed for each residue along the whole MD simulation.

Supplementary Table 5. Secondary structure components, mean and standard deviation values among the PHF6 MD simulation.

Secondary Structure	MD mean values
Coil	33
β-Sheet	50
β-Bridge	0
Bend	0
3-Helix	0
Turn	0

Supplementary Table 6. Center of mass distances (nm) of the C alpha atoms of each fibril layer versus the successive, mean and standard deviation values among the PHF6 MD simulation

Layer	Mean	SD
1-2	0.377	0.003
2-3	0.377	0.003
3-4	0.377	0.003
4-5	0.378	0.003
5-6	0.378	0.003
6-7	0.378	0.003
7-8	0.377	0.003
8-9	0.378	0.003
9-10	0.378	0.003
10-11	0.378	0.003
11-12	0.377	0.003

Supplementary Note 11.4. Tau441 fibrils and Tau441 macromolecular architecture

Molecular dynamic simulations were performed starting from the Cryo-EM atomic models of paired helical (*p*-helical) and straight Tau filaments (*s*-filaments), recently purified and characterized from AD brain by Fitzpatrick (Cryo-EM).[50] These Tau441 morphologies are both composed by residues 306-378 of Tau protein, which represent the seed regions of Tau aggregation. The residues arrange into combined cross-β and β-helix structures to form a double helical stack of C-shaped subunits. The *p*-helical and *s*-filaments supermolecular inter-protofilament packings differ in the lateral contacts between two fibril subunits each composed by 5 layers of Tau441 core regions (**Supplementary Figure 19**).

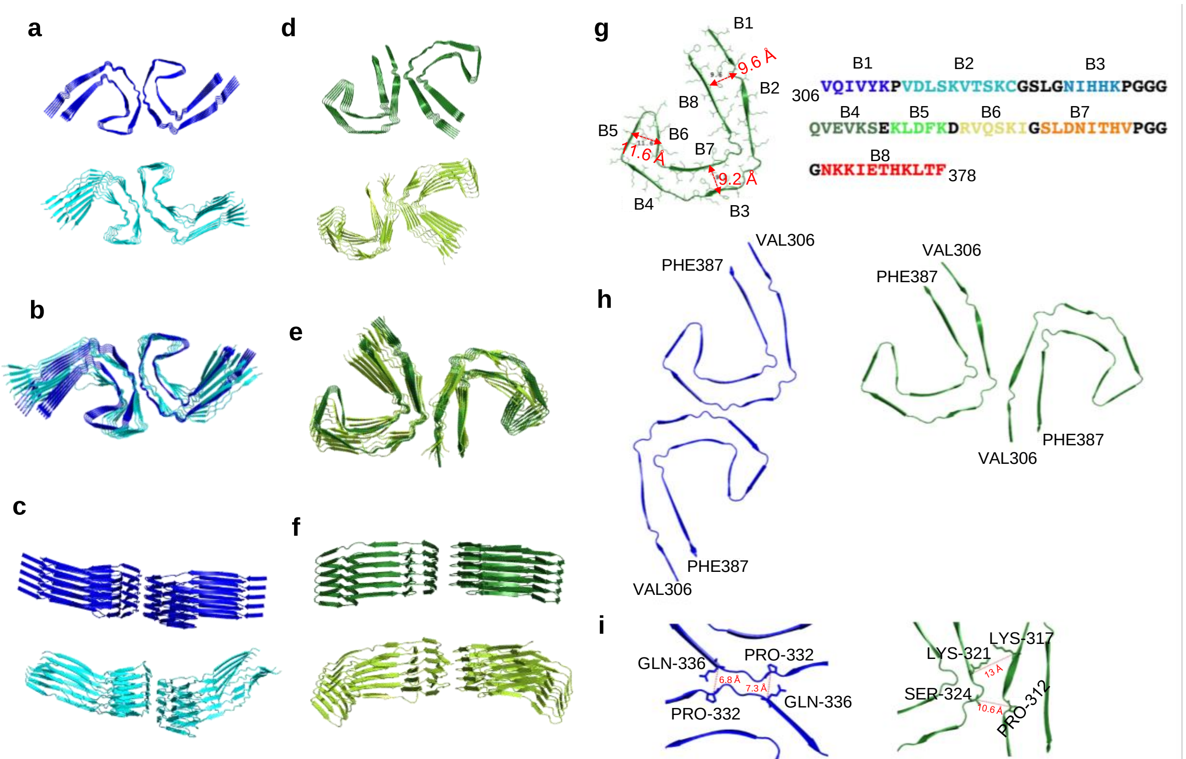

Supplementary Figure 19 Starting PDB and our model structures of *p*-helical and *s*-filaments Tau441 morphologies. a-c, PDB (blue) and last MD frame (cyan) of p-helical tau from three different views. **d-f**, PDB (dark green) and last MD frame (light green) of s-filaments tau from three different views. **g**, Tau441 core sequence used in our models, the eight β-strands forming the β sheet regions are indicated and differentially colored. **h**, first pairs of p-helical (blue) and s-filaments (green) interacting subunits are shown in cartoon. **i**, zoomed view of inter-molecular contacts. The heavy atoms of those residues delimiting the subunit interfaces are shown in sticks, red dashed lines are used to indicate the C alpha residue distances.

Supplementary Note 11.5 Molecular Dynamic simulations of Tau441 fibril

Both the systems keep the overall macromolecular arrangement after 30 ns of MD simulation. The chain RMSD profiles suggest that the *p*-helical goes to greater rearrangements compared to the *s*-filaments morphology, (**Supplementary Table 7** and **Supplementary Figure 20**). In details, the *p*-helical termini regions, chain 1, 2 and 10, show RMSD mean values with the respect to the starting conformation of 6.1, 5.7 and 5.8 Å, respectively. The β bridges shown in the starting *p*-helical and s-filaments structures are quickly lost during the simulation, but the percentage of residues involved in coil and beta structures are globally preserved, particularly for *s*-filaments (**Supplementary Table 8**). Indeed, the *p*-helical morphology shows a partial loss of the β components, which involve the 67% of residues in the reference structure but only the 54% during trajectory. On the contrary, in the *s*-filaments morphology the percentage of beta residues in the reference structure (63%) is consistent with the mean value among the simulation (61%, **Supplementary Table 8** and **Supplementary Figure 20**). The Cα-Cα distance of successive V309 residues along the fibril, result hold for both morphologies (**Supplementary Table 9**) during the MD runs. These measurements represent the inter β strand spaces and are consistent with the picks at ~4.8 Å observed in the XRD spectra. Only a partial, but not significant, detachment (around 15-20 ns of simulation time) of chain 8 versus chain 10 in *p*-helical proto-filament occurs after 15 ns (**Supplementary Figure 23**). Although this behavior is not affecting the global assessment of the *p*-helical stability, is consistent with the higher RMSD values and the partial loss of beta components, confirming a minor stability of this morphology respect to the *s*-filaments. For both morphologies no major fibril perturbations occur during our simulations and the global fibril compactness is maintained as shown in **Supplementary Figure 19a-f**. In both the Tau441 isoforms the intra-molecular distances between two facing sheets, within the same chain, result approximately 9 Å along the trajectories, in close agreement with the pick values around 9.4 Å registered by XRD measurements (**Supplementary Table 11**). The inter-molecular distances between facing subunits, at the interfaces between two lateral Tau441 chains, result

approximately 12 Å along both the trajectories (**Supplementary Table 10**). This measure results rather fluctuating along the simulations and likely correspond to the XRD 12.5 Å picks registered at higher temperature.

Our simulations suggest a higher variability of inter-molecular with the respect to the intra-molecular distances (standard deviations in **Supplementary Table 10** and **Supplementary Table 11**, respectively), likely explaining the smaller pick density at 12.5 Å of XRD measurement. Indeed, these connections are responsible of supermolecular architecture which represents a second step of aggregation, weaker than the intra-molecular interactions. MD trajectories suggest that the s-filaments proto-filament subunit interface confers greater global stability than that in p-helical arrangement. The simulations could clarify the pick at 12.5 Å of XRD measurement registered only at high temperature, suggesting that the laminar convective flow induces the ordered molecular assembling, and that the fiber growth formed at high temperature preferentially arrange in the more stable s-filament morphology.

Our findings reveal that in the *p*-helical filaments some structural elements are less stable than the *s*-filaments morphologies. The *s*-filaments proto-filament subunit interface confers greater stability than that in *p*-helical arrangement, but for both systems no major fibril perturbations occur during our simulations and the global fibril compactness is maintained as shown in **Supplementary Figure 19**.

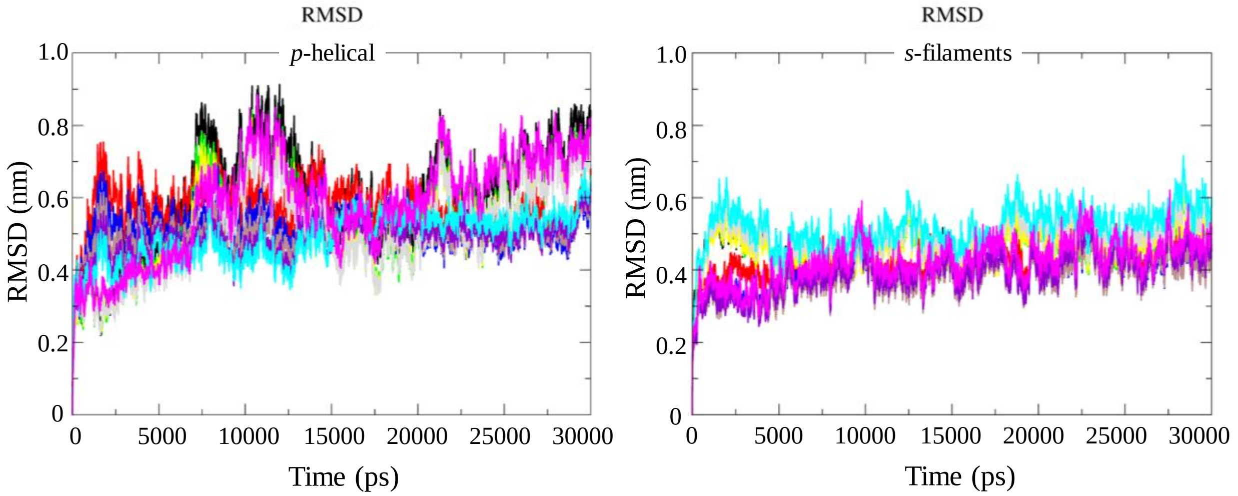

Supplementary Figure 20 RMSD profiles computed for each fibril chain along the Tau441 MD simulation.

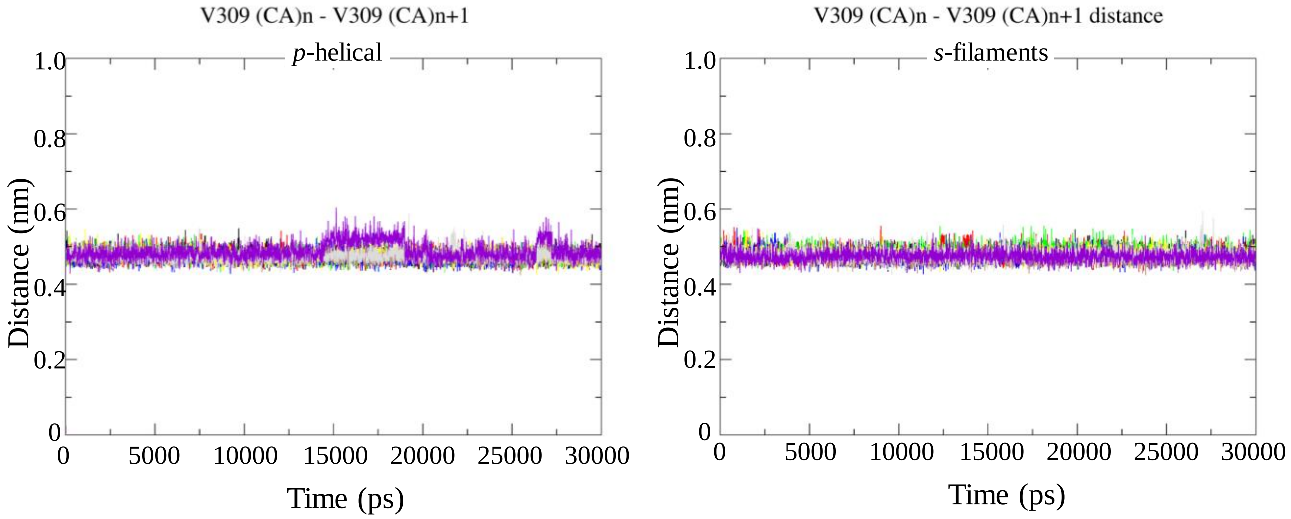

Supplementary Figure 21 Center of mass distances of the C alpha atoms of each V309 residue versus the successive along the *p*-helical and *s*-filaments simulations.

Supplementary Table 7. Center of mass distances of the C alpha atoms (RMSD in nanometer) of each fibril layer versus the successive, mean and standard deviation values among the Tau441 MD simulation.

Chain	*p*-helical		*s*-filaments	
	Mean	SD	Mean	SD
1	0.61	0.15	0.47	0.042
2	0.57	0.06	0.43	0.045
3	0.55	0.14	0.46	0.044
4	0.52	0.06	0.39	0.047
5	0.53	0.13	0.46	0.047
6	0.50	0.05	0.37	0.051
7	0.52	0.13	0.48	0.052
8	0.49	0.05	0.39	0.058
9	0.49	0.07	0.52	0.056
10	0.58	0.14	0.42	0.061

Supplementary Table 8. Secondary structure components ratio (%) of Tau441 systems among the MD simulation.

	MD mean values		PDB	
Secondary Structure	*p*-helical	*s*-filaments	*p*-helical (5o3l)	*s*-filaments (5o3t)
Coil	37	30	28	26
β-Sheet	54	61	65	63
β-Bridge	1	0	19	17
Bend	7	6	1	9
Turn	0	1	1	9

Supplementary Table 9. C alpha atom distances of each V309 residues in Tau441 systems versus the successive, mean and standard deviation values among the MD simulation.

V309 (CA)n-V309(CA)n+1 distance (nm)				
Chain	*p*-helical		*s*-filaments	
	Mean	SD	Mean	SD
1-3	0.47	0.01	0.48	0.01
2-4	0.47	0.01	0.48	0.02
3-5	0.47	0.01	0.49	0.02
4-6	0.47	0.01	0.47	0.01
5-7	0.48	0.01	0.48	0.01
6-8	0.47	0.01	0.47	0.01
7-9	0.48	0.02	0.48	0.02
8-10	0.49	0.02	0.47	0.01

Supplementary Table 10. Inter-subunits distances in *p*-helical and *s*-filaments isoforms. The C alpha distances between those residues delimiting the subunit interfaces in both isoforms are reported as mean values along the simulations.

**Inter subunits residue distances (Å)**			
***p*-helical**	chains	**Mean**	**SD**
**P332-Q336**	1-2	11.6	0.6
	3-4	11.6	0.4
	5-6	11.7	0.3
	7-8	11.6	0.3
	8-9	12.0	0.4
**Q336-P332**	1-2	12.1	0.3
	3-4	12.2	0.2
	5-6	12.0	0.2
	7-8	12.2	0.3
	8-9	11.6	0.4
**Mean**		11.8	0.3
**s-filaments**	chains	**Mean**	**SD**
**S324-P312**	1-2	9.5	0.7
	3-4	10.9	0.7
	5-6	11.5	0.7
	7-8	11.2	0.5
	8-9	11.4	0.6
**K317-K321**	1-2	13.4	0.8
	3-4	12.6	0.5
	5-6	12.1	0.4
	7-8	11.6	0.4
	8-9	11.5	0.7
**Mean**		11.6	0.6

Supplementary Table 11. C alpha atom distances of β-sheet pairs of Tau441 systems, mean and standard deviation values among the MD simulation.

	***p*-helical**	***s*-filaments**	***p*-helical**	***s*-filaments**
	**Mean (Å)**		**SD**	
**B12-B8**	9.5	9.4	0.6	0.6
	9.3	8.6	0.9	0.5
	9.3	8.0	0.4	0.6
	9.5	9.0	0.5	0.3
	9.4	9.1	0.4	0.6
	9.8	9.4	0.4	0.3
	9.8	9.5	0.5	0.4
	9.9	9.6	0.4	0.4
	9.7	9.5	0.6	0.4
	6.7	6.7	1.3	0.6
**B3-B7**	8.9	8.0	0.4	0.6
	8.5	7.9	0.6	0.7
	7.8	8.9	0.7	0.6
	8.7	8.6	0.5	0.5
	8.4	8.7	0.5	0.3
	9.1	8.6	0.4	0.4
	9.0	9.0	0.4	0.4
	8.9	8.8	0.4	0.4
	9.1	8.9	0.3	0.4
	8.5	8.9	0.7	0.5
**B5-B6**	9.5	8.5	0.4	0.6
	8.6	9.4	0.7	0.4
	8.6	8.8	0.6	0.4
	8.9	8.7	0.6	0.3
	8.5	9.0	0.3	0.3
	8.9	8.9	0.4	0.3
	8.9	8.8	0.3	0.4
	9.1	8.9	0.3	0.3
	9.1	8.8	0.3	0.4
	9.1	8.4	0.4	0.7
**mean**	9.0	8.8	0.5	0.5

References

1. Hill, E. K., Krebs, B., Goodall, D. G., Howlett, G. J. & Dunstan, D. E. Shear flow induces amyloid fibril formation. *Biomacromolecules* **7**, 10-13, (2006).
2. McBride, S. A., Sanford, S. P., Lopez, J. M. & Hirsa, A. H. Shear-induced amyloid fibrillization: the role of inertia. *Soft Matter* **12**, 3461-3467, (2016).
3. Yuan, T. Z. *et al.* Shear-stress-mediated refolding of proteins from aggregates and inclusion bodies. *Chembiochem* **16**, 393-396, (2015).
4. Moretti, M. *et al.* Confined laminar flow on a super-hydrophobic surface drives the initial stages of tau protein aggregation. *Microelectron Eng* **191**, 54-59, (2018).
5. Marini, M. *et al.* The structure of DNA by direct imaging. *Sci Adv* **1**, e1500734, (2015).
6. De Angelis, F. *et al.* Breaking the diffusion limit with super-hydrophobic delivery of molecules to plasmonic nanofocusing SERS structures. *Nat Photonics* **5**, 682-687, (2011).
7. Ristenpart, W. D., Kim, P. G., Domingues, C., Wan, J. & Stone, H. A. Influence of substrate conductivity on circulation reversal in evaporating drops. *Phys Rev Lett* **99**, 234502, (2007).
8. Lu, G., Duan, Y. Y., Wang, X. D. & Lee, D. J. Internal flow in evaporating droplet on heated solid surface. *Int J Heat Mass Tran* **54**, 4437-4447, (2011).
9. Pan, Z., Dash, S., Weibel, J. A. & Garimella, S. V. Assessment of water droplet evaporation mechanisms on hydrophobic and superhydrophobic substrates. *Langmuir* **29**, 15831-15841, (2013).
10. Tam, D., von Arnim, V., McKinley, G. H. & Hosoi, A. E. Marangoni convection in droplets on superhydrophobic surfaces. *J Fluid Mech* **624**, 101-123, (2009).
11. Bintein, P.-B., Bense, H., Clanet, C. & Quéré, D. Self-propelling droplets on fibres subject to a crosswind. *Nature Physics* **15**, 1027-1032, (2019).
12. Cini, R., Loglio, G. & Ficalbi, A. Temperature dependence of the surface tension of water by the equilibrium ring method. *Journal of Colloid and Interface Science* **41**, 287-297, (1972).
13. Savino, R., Cecere, A. & Di Paola, R. Surface tension-driven flow in wickless heat pipes with self-rewetting fluids. *Int J Heat Fluid Fl* **30**, 380-388, (2009).
14. Morozova-Roche, L. A. *et al.* Amyloid fibril formation and seeding by wild-type human lysozyme and its disease-related mutational variants. *J Struct Biol* **130**, 339-351, (2000).
15. Moretti, M. *et al.* Raman study of lysozyme amyloid fibrils suspended on super-hydrophobic surfaces by shear flow. *Microelectron Eng* **178**, 194-198, (2017).
16. Chamberlain, A. K. *et al.* Ultrastructural organization of amyloid fibrils by atomic force microscopy. *Biophys J* **79**, 3282-3293, (2000).
17. Moretti, M. *et al.* AFM characterization of biomolecules in physiological environment by an advanced nanofabricated probe. *Microsc Res Techniq* **75**, 1723-1731, (2012).
18. Spillantini, M. G. & Goedert, M. Tau protein pathology in neurodegenerative diseases. *Trends Neurosci* **21**, 428-433, (1998).
19. Goedert, M., Eisenberg, D. S. & Crowther, R. A. Propagation of Tau Aggregates and Neurodegeneration. *Annu Rev Neurosci* **40**, 189-210, (2017).
20. Fitzpatrick, A. W. P. *et al.* Cryo-EM structures of tau filaments from Alzheimer's disease. *Nature* **547**, 185-+, (2017).
21. Clavaguera, F. *et al.* Transmission and spreading of tauopathy in transgenic mouse brain. *Nat Cell Biol* **11**, 909-U325, (2009).
22. Gerson, J. E., Sengupta, U. & Kayed, R. in *Tau Protein: Methods and Protocols* (ed Caroline Smet-Nocca) 141-157 (Springer New York, 2017).
23. Goedert, M. The ordered assembly of tau is the gain-of-toxic function that causes human tauopathies. *Alzheimers Dement* **12**, 1040-1050, (2016).

24 Scholz, T. & Mandelkow, E. Transport and diffusion of Tau protein in neurons. *Cell Mol Life Sci* **71**, 3139-3150, (2014).
25 He, Z. H. *et al.* Amyloid-beta plaques enhance Alzheimer's brain tau-seeded pathologies by facilitating neuritic plaque tau aggregation. *Nat Med* **24**, 29-+, (2018).
26 Fichou, Y. *et al.* The elusive tau molecular structures: can we translate the recent breakthroughs into new targets for intervention? *Acta Neuropathol Commun* **7**, 31-31, (2019).
27 Sibille, N. *et al.* Structural Impact of Heparin Binding to Full-Length Tau As Studied by NMR Spectroscopy. *Biochemistry-Us* **45**, 12560-12572, (2006).
28 Bordoli, L., Kiefer, F. & Schwede, T. Assessment of disorder predictions in CASP7. *Proteins* **69**, 129-136, (2007).
29 Dyson, H. J. & Wright, P. E. Unfolded proteins and protein folding studied by NMR. *Chem Rev* **104**, 3607-3622, (2004).
30 Bernado, P. & Svergun, D. I. Structural analysis of intrinsically disordered proteins by small-angle X-ray scattering. *Molecular Biosystems* **8**, 151-167, (2012).
31 Ganguly, P. *et al.* Tau Assembly: The Dominant Role of PHF6 (VQIVYK) in Microtubule Binding Region Repeat R3. *Journal of Physical Chemistry B* **119**, 4582-4593, (2015).
32 Sawaya, M. R. *et al.* Atomic structures of amyloid cross-beta spines reveal varied steric zippers. *Nature* **447**, 453-457, (2007).
33 Perez, M. *et al.* The role of the VQIVYK peptide in tau protein phosphorylation. *Journal of Neurochemistry* **103**, 1447-1460, (2007).
34 Altamura, D. *et al.* X-ray microimaging laboratory (XMI-LAB). *J Appl Crystallogr* **45**, 869-873, (2012).
35 Sibillano, T. *et al.* Interfibrillar packing of bovine cornea by table-top and synchrotron scanning SAXS microscopy. *J Appl Crystallogr* **49**, 1231-1239, (2016).
36 Siliqi, D. *et al.* SUNBIM: a package for X-ray imaging of nano- and biomaterials using SAXS, WAXS, GISAXS and GIWAXS techniques. *J Appl Crystallogr* **49**, 1107-1114, (2016).
37 Berhanu, W. M. & Hansmann, U. H. E. in *Advances in Protein Chemistry and Structural Biology* Vol. 96 (ed Tatyana Karabencheva-Christova) 113-141 (Academic Press, 2014).
38 Diaferia, C. *et al.* Assembly modes of hexaphenylalanine variants as function of the charge states of their terminal ends. *Soft Matter* **14**, 8219-8230, (2018).
39 Naldi, M. *et al.* Amyloid beta-peptide 25-35 self-assembly and its inhibition: a model undecapeptide system to gain atomistic and secondary structure details of the Alzheimer's disease process and treatment. *ACS Chem Neurosci* **3**, 952-962, (2012).
40 Chawla, M., Autiero, I., Oliva, R. & Cavallo, L. Energetics and dynamics of the non-natural fluorescent 4AP:DAP base pair. *Phys Chem Chem Phys* **20**, 3699-3709, (2018).
41 Autiero, I., Ruvo, M., Improta, R. & Vitagliano, L. The intrinsic flexibility of the aptamer targeting the ribosomal protein S8 is a key factor for the molecular recognition. *Biochim Biophys Acta Gen Subj* **1862**, 1006-1016, (2018).
42 Barone, D., Balasco, N., Autiero, I. & Vitagliano, L. The dynamic properties of the Hepatitis C Virus E2 envelope protein unraveled by molecular dynamics. *J Biomol Struct Dyn* **35**, 805-816, (2017).
43 Mark, P. & Nilsson, L. Structure and dynamics of the TIP3P, SPC, and SPC/E water models at 298 K. *Journal of Physical Chemistry B* **105**, 24a-24a, (2001).
44 Mackerell, A. D., Jr., Feig, M. & Brooks, C. L., 3rd. Extending the treatment of backbone energetics in protein force fields: limitations of gas-phase quantum mechanics in reproducing protein conformational distributions in molecular dynamics simulations. *J Comput Chem* **25**, 1400-1415, (2004).
45 MacKerell, A. D. *et al.* All-Atom Empirical Potential for Molecular Modeling and Dynamics Studies of Proteins. *The Journal of Physical Chemistry B* **102**, 3586-3616, (1998).

46 Hess, B., Kutzner, C., van der Spoel, D. & Lindahl, E. GROMACS 4: Algorithms for highly efficient, load-balanced, and scalable molecular simulation. *Journal of Chemical Theory and Computation* **4**, 435-447, (2008).

47 Autiero, I., Saviano, M. & Langella, E. In silico investigation and targeting of amyloid β oligomers of different size. *Molecular BioSystems* **9**, 2118-2124, (2013).

48 Autiero, I., Langella, E. & Saviano, M. Insights into the mechanism of interaction between trehalose-conjugated beta-sheet breaker peptides and Aβ(1–42) fibrils by molecular dynamics simulations. *Molecular BioSystems* **9**, 2835-2841, (2013).

49 KrishnaKumar, V. G., Paul, A., Gazit, E. & Segal, D. Mechanistic insights into remodeled Tau-derived PHF6 peptide fibrils by Naphthoquinone-Tryptophan hybrids. *Sci Rep* **8**, 71, (2018).

50 Fitzpatrick, A. W. P. *et al.* Cryo-EM structures of tau filaments from Alzheimer's disease. *Nature* **547**, 185, (2017).
