# Supplementary material for: A droplet reactor on a super-hydrophobic surface allows control and characterization of amyloid fibril growth
Source: Commun Biol. 2020 Aug 20;3:457. doi: 10.1038/s42003-020-01187-7 (PMC7441408; doi:10.1038/s42003-020-01187-7)
Supplement: Supplementary file 6 — Description of Additional Supplementary Files [file 42003_2020_1187_MOESM6_ESM.pdf]

Description of Additional Supplementary Files

File Name: **Supplementary Movie 1**
Description: Convection in droplet on SHS with various temperature gradients

File Name: **Supplementary Movie 2**
Description: Confined convective flow in droplet on SHS

File Name: **Supplementary Movie 3**
Description: Lysozyme amyloid fibrils growth driven by confined convective flow in droplet on SHS

File Name: **Supplementary Movie 4**
Description: Ultra-fast imaging reveals droplet depinning dynamics
