# Supplementary material for: A droplet reactor on a super-hydrophobic surface allows control and characterization of amyloid fibril growth
Source: Commun Biol. 2020 Aug 20;3:457. doi: 10.1038/s42003-020-01187-7 (PMC7441408; doi:10.1038/s42003-020-01187-7)
Supplement: Supplementary file 7 — Reporting Summary [file 42003_2020_1187_MOESM7_ESM.pdf]

nature research

Corresponding author(s): Enzo Di Fabrizio

Last updated by author(s): Jul 12, 2020

Reporting Summary

Nature Research wishes to improve the reproducibility of the work that we publish. This form provides structure for consistency and transparency in reporting. For further information on Nature Research policies, see Authors & Referees and the Editorial Policy Checklist.

Please do not complete any field with "not applicable" or n/a. Refer to the help text for what text to use if an item is not relevant to your study.
For final submission: please carefully check your responses for accuracy; you will not be able to make changes later.

Statistics

For all statistical analyses, confirm that the following items are present in the figure legend, table legend, main text, or Methods section.

n/a	Confirmed	
☐	☒	The exact sample size (n) for each experimental group/condition, given as a discrete number and unit of measurement
☐	☒	A statement on whether measurements were taken from distinct samples or whether the same sample was measured repeatedly
☒	☐	The statistical test(s) used AND whether they are one- or two-sided<br>*Only common tests should be described solely by name; describe more complex techniques in the Methods section.*
☒	☐	A description of all covariates tested
☒	☐	A description of any assumptions or corrections, such as tests of normality and adjustment for multiple comparisons
☒	☐	A full description of the statistical parameters including central tendency (e.g. means) or other basic estimates (e.g. regression coefficient) AND variation (e.g. standard deviation) or associated estimates of uncertainty (e.g. confidence intervals)
☒	☐	For null hypothesis testing, the test statistic (e.g. F, t, r) with confidence intervals, effect sizes, degrees of freedom and P value noted<br>*Give P values as exact values whenever suitable.*
☒	☐	For Bayesian analysis, information on the choice of priors and Markov chain Monte Carlo settings
☒	☐	For hierarchical and complex designs, identification of the appropriate level for tests and full reporting of outcomes
☒	☐	Estimates of effect sizes (e.g. Cohen's d, Pearson's r), indicating how they were calculated

Our web collection on statistics for biologists contains articles on many of the points above.

Software and code

Policy information about availability of computer code

Data collection

1. HCImage is used to control ORCA-Flash Camera to collect droplet fluorescent imaging data;
2. IRBS3 is used to collect Thermal images;
3. ANSYS®, Release 19.1 (Fluent solver) is used for convection simulation;
4. WITec Suite 5.2 was used to collect laser confocal Raman data;
5. APEX3 software is used to control 2D-XRD data collection;
6. GROMACS (5.1.2) and Pymol packages (http://www.pymol.org) are used for MD modeling; 6. JPK proprietary software for AFM data collection;
7. LabView is used for temperature control;
8. PFV software is used for ultra-fast imaging collection;
9. SolidWorks software is used for imaging platform design and modeling;
10. SAXSGUI received with SMAX3000 Rigaku machine is used for SAXS/WAXS data collection.
11. Tecnai Microscope Control program is used for TEM imaging.

Data analysis	1. ImageJ Trackmake plug-in is used for droplet convection profile analysis;<br>2. IRBS3 is used to measure temperature profiles in droplet;<br>3. ANSYS®, Release 19.1 (Fluent solver) is used for simulation data analysis;<br>4. WITec Suite 5.2 and WiRE 3.2 are used for Raman spectrum analysis;<br>5. DIFFRAC.EVA from Bruker is used to analysis 2D-XRD data;<br>6. JPK proprietary software for AFM data analysis;<br>7. Origin Pro and Excel are used to plot Raman and XRD profiles;<br>8. GROMACS (5.1.2) and Pymol packages are used for MD analysis;<br>9. SUNBIM (http://www.ba.ic.cnr.it/softwareic/sunbimweb/) was used for SAXS/WAXS data analysis.<br>10. GMS3 software is used for TEM images analysis.

For manuscripts utilizing custom algorithms or software that are central to the research but not yet described in published literature, software must be made available to editors/reviewers. We strongly encourage code deposition in a community repository (e.g. GitHub). See the Nature Research guidelines for submitting code & software for further information.

Data

Policy information about availability of data

All manuscripts must include a data availability statement. This statement should provide the following information, where applicable:

- Accession codes, unique identifiers, or web links for publicly available datasets
- A list of figures that have associated raw data
- A description of any restrictions on data availability

Fig. 4b, Fig. 5a-5i, Figs. 6c-6e, Figs. 7c-7i have associated raw data. And these data are available from Dryad through the following link: https://datadryad.org/stash/share/UpN68kYKnBxs4z5iPvGo9-HbybXiXtfTD0Kc7KKfwo8

Field-specific reporting

Please select the one below that is the best fit for your research. If you are not sure, read the appropriate sections before making your selection.

☒ Life sciences ☐ Behavioural & social sciences ☐ Ecological, evolutionary & environmental sciences

Life sciences study design

All studies must disclose on these points even when the disclosure is negative.

Sample size	Our measurements on prepared samples are based on basic data statistics. The multiple (more than 10) samples with prepared protein fibers are measured by Raman, XRD and WAXS/SAXS for data statistics.
Data exclusions	N/A
Replication	The convection profiles in droplet reactor and protein fibers formation on SHS shows great reproducibility, multiple (more than 10) samples are prepared with our methods.
Randomization	N/A
Blinding	N/A

Reporting for specific materials, systems and methods

We require information from authors about some types of materials, experimental systems and methods used in many studies. Here, indicate whether each material, system or method listed is relevant to your study. If you are not sure if a list item applies to your research, read the appropriate section before selecting a response.

Materials & experimental systems

n/a	Involved in the study	
☒	☐	Antibodies
☒	☐	Eukaryotic cell lines
☒	☐	Palaeontology
☒	☐	Animals and other organisms
☒	☐	Human research participants
☒	☐	Clinical data

Methods

n/a	Involved in the study	
☒	☐	ChIP-seq
☒	☐	Flow cytometry
☒	☐	MRI-based neuroimaging

This checklist template is licensed under a Creative Commons Attribution 4.0 International License, which permits use, sharing, adaptation, distribution and reproduction in any medium or format, as long as you give appropriate credit to the original author(s) and the source, provide a link to the Creative Commons license, and indicate if changes were made. The images or other third party material in this article are included in the article's Creative Commons license, unless indicated otherwise in a credit line to the material. If material is not included in the article's Creative Commons license and your intended use is not permitted by statutory regulation or exceeds the permitted use, you will need to obtain permission directly from the copyright holder. To view a copy of this license, visit http://creativecommons.org/licenses/by/4.0/
